# Supplementary material for: Inferring cell state by quantitative motility analysis reveals a dynamic state system and broken detailed balance
Source: PLoS Comput Biol. 2018 Jan 16;14(1):e1005927. doi: 10.1371/journal.pcbi.1005927 (PMC5786322; doi:10.1371/journal.pcbi.1005927)
Supplement: S1 Text — (PDF) [file pcbi.1005927.s001.pdf]

Supplemental Methods

Inferring cell state by quantitative motility analysis reveals a dynamic state system and broken detailed balance

Jacob C. Kimmel, Amy Y. Chang, Andrew S. Brack, Wallace F. Marshall

Animals

All mice were housed at the University of California San Francisco following UCSF Institutional Use and Care of Animals Committee guidelines. Adult male C57Bl/6 mice (2-4 m.o.) were used for all myogenic cell experiments. Adult female C57Bl/6 mice (2-3 m.o.) were used as mothers to derive MEFs.

Myoblast and Muscle Stem Cell Isolations

Mononuclear cells were isolated from limb muscles of adult mice as described (Shea et al. 2010). Myoblasts were isolated by negative-selection against *Sca1* using the EasySep Endothelial Selection kit (Stem Cell Tech., Vancouver, BC) and subsequent 5 minute preplating. Myoblasts were maintained in myogenic growth media (F10, 20% FBS, and [5 ng/mL] FGF2) on sarcoma-derived ECM (Sigma, St. Louis, MO) coated dishes. All myoblasts used in experiments were below passage 4.

Wild-type muscle stem cells were isolated by FACS after incubating the bulk mononuclear cell population with anti-VCAM-PE (BioLegend, San Diego, CA), anti-α7-integrin-649 (R&D Systems, Minneapolis, MN), anti-CD31-PE-Cy7 (BD Pharmingen, San Jose, CA), anti-CD45-PE-Cy7 (BD Pharmingen, San Jose, CA), and anti-Sca1-APC-Cy7 (BD Pharmingen, San Jose, CA). Propidium iodide was added as a live/dead marker. Muscle stem cells were selected based on a $VCAM^+/\alpha7\text{-integrin}^+$, $CD31^-/CD45^-/Sca1^-/PI^-$ profile, as previously described (Chakkalakal et al. 2014).

MEF Cell Culture

Wild-type MEFs were isolated from E13.5 embryos as described (E Michalska 2007). MEFs isolated from two separate mothers were used for these experiments. Transformed MEFs were generated as described (Truitt et al. 2015), generously donated by the authors. All wild-type MEFs used in experiments were from passages 2 - 5. All MEFs were maintained in DMEM, 10% FBS, 1% Penicillin / Streptomycin at 37° C and 5% CO_2.

Timelapse Motility Imaging

All imaging was performed on a Nikon Ti using a 20X air objective and an Oko temperature and carbon dioxide incubation unit set to 37° C and 5% CO_2. Images were captured by a Hamamatsu C11440-22CU camera (pixel size 6.5 x 6.5 μm). Twenty-five images with 10% edge overlap covering a 3.3 mm square at the center of each 96 well were captured. Focus was maintained throughout the timelapse using the Nikon PerfectFocus system. Each XY position was segmented and analyzed independently. XY positions that experienced overexposure or out of focus events were removed from analysis by manual screening of the data.

MuSCs were seeded at 500-1000 cells/well in 20 wells of a 96 well plate immediately after FACS sorting. After 23 hours to allow for adaptation to culture, media was exchanged for the relevant experimental medium and MuSCs were incubated for 1 hour to adapt. MuSCs were imaged at 20X in DIC for 10 hours with a temporal resolution of 6.5 minutes / frame. Myoblasts were passaged and plated on sarcoma-derived ECM coated optically clear plastic 96 well plates 24 hours prior to imaging at 300-500 cells/well. Media exchanges were performed 1 hr prior to imaging, in the same manner as MuSCs. Myoblast imaging was performed at 20X in DIC for 10 hours with a temporal resolution of 6.5 minutes / frame. For FGF2 perturbation experiments,

10 wells of a 96 well plate were imaged in growth media with [5 ng/mL] FGF2, and the remaining 10 were imaged in growth media with [0 ng/mL] FGF2. MEFs were similarly plated in optically clear plastic 96 well plates (without ECM coating) 16-20 hours prior to imaging at the 300-500 cells/well and imaged in the same manner. The initial 18 frames of imaging were omitted from downstream analysis for MEF and myoblast experiments, and the initial 30 from MuSC experiments to minimize the effect of initial XY drift in our imaging apparatus.

Image Segmentation

Brightfield images were segmented using custom segmentation algorithms, optimized for each of the systems we investigated. Segmentation algorithms are steerable filters (Jacob and Unser 2004) for edge detection, top-hat filtering ("*Image Analysis and Mathematical Morphology*," n.d.), and Otsu thresholding (Otsu 1975), utilizing implementations available in the Matlab Image Processing Toolbox. These standard segmentation methods were combined with contrast adjustments, noise filtration, and background subtraction as needed for each system. Images were segmented independently for each field-of-view. Cells spanning multiple fields of view were not segmented. For tracking, the centroid of segmented cells was taken to be the cell location.

Heteromotility

All code for the Heteromotility tool and downstream analysis is available on the Heteromotility Github page and in the Python Package Index (PyPI) (https://github.com/cellgeometry/heteromotility).

For all analysis performed in this manuscript, all features except the turning features were utilized. The turning features are not considered for any of our analyses.

Cell Tracking

Tracking was of cell centroids was performed using a global nearest neighbor optimization approach, as implemented in uTrack (Jaqaman et al. 2008). Specific parameters were devised for each cell type to optimize tracking, and are provided in the Heteromotility Github code base. Only cells that remained in the same field-of-view throughout the timelapse were tracked. All tracks were visually screened for accuracy, and any erroneous tracking events (tracking of non-cellular objects, tracks crossing, etc.) were removed from analysis.

Distance and Speed Calculations

Total distance is calculated as the sum of all displacements along a given cell's path. Net distance is the distance between the initial and final points along the path. Progressivity is defined as the ratio of net distance to total distance traveled.

Minimum speed is the minimum displacement value along a cell's path. Maximum speed is the maximum displacement value. Average speed is the mean of all displacement values. Time Moving is the proportion of time a cell spends above a given threshold speed. Average moving speed is the mean speed of a cell while above a threshold value.

Turning Features

To determine the magnitude and direction of a cell's turning behavior, the cell's instantaneous direction is first established by performing linear regression on a range of points size 2δ about every point p_t in the time series T where $\delta < t < T - \delta$. The cell's movement direction relative to the x axis on the interval $[p_t - \delta, p_t + \delta]$ is considered to establish directionality to the regression. This vector R is assumed to represent the cell's

direction at p_t. The cell's position $p_t + \tau$ is considered for a given time lag τ. If $p_t + \tau$ falls left relative to R, the cell is said to have made a left turn, and vice versa. The magnitude of each turn is calculated as the angle between the vector R and the vector from p_t to $p_t + tau$.

Correlation Features

The linearity of a cell's path is calculated as Pearson's r^2 value of a linear regression through all two-dimensional positions a cell occupied during the time course. Similarly, the monotonicity of a cell path is calculated as Spearman's ρ^2 for the same distribution of two-dimensional coordinates.

Displacement Distribution Features

Mean Squared Displacement Coefficient

Mean squared displacement (MSD) is calculated for the two-dimensional vector of positions X and a given time lag τ as

$$MSD(\tau) = \langle x_{t+\tau} - x_t \rangle$$

Here, τ denotes the given time lag, x_t denotes the position at time t, and $\langle\rangle$ denotes the mean value of an expression. MSD is calculated for τ in the range $[1, 30]$. The relationship of MSD to τ can be expressed in a general form $MSD(\tau) \propto \tau^\alpha$. The exponent α in this relationship can be easily estimated by power transformation of the resulting values, such that $\log MSD \propto \alpha \log \tau$ and α not represents the slope of a linear regression through $\log MSD$ vs $\log \tau$.

Kurtosis

To calculate the kurtosis of a cell's displacements, we consider displacement sizes for a variable time lag τ, so that X_τ represents the distribution of displacements for a given time lag τ. We calculate excess kurtosis as the standard fourth central moment:

$$kurtosis(X_\tau) = \langle x^4 - \bar{x} \rangle - 3$$

Non-Gaussian Parameter

The Non-Gaussian parameter α_2 is calculated as described (Rahman 1964).

$$\alpha_2 = \frac{\langle \Delta x^4 \rangle}{3 \langle \Delta x^2 \rangle^2} - 1$$

Here, $\langle\rangle$ denotes the mean of an expression and Δx denotes an individual displacement in the distribution. Effectively, the α_2 parameter denotes a ratio of kurtosis to variance for a distribution, as $kurtosis(X) = \langle x^4 - \bar{x} \rangle$, and $\sigma^2 = \langle x - \bar{x} \rangle^2$. For a Gaussian distribution, $\alpha_2 = 0$. As the tails of a distribution increase in weight, α_2 also increases. As such, α_2 acts as a metric of the Non-Gaussian nature of a given distribution, with higher α_2 values indicating that the distribution is less Gaussian and heavier tailed.

Self-Similarity Metrics

Hurst Exponent Estimation

Fractal Brownian motion (fBm) is a more general description of Brownian motion that allows for interdependence between displacements (B B Mandelbrot and Van Ness 1968). fBm is defined as a random Gaussian process parameterized by H:

$$B_H(t) = B_H(0) + \frac{1}{\Gamma(H+\frac{1}{2})}\int_{-\infty}^{0}[(t-s)^H - \frac{1}{2} - (-s)^H - \frac{1}{2}]dB(s) + \int_{0}^{t}[(t-s)^H - \frac{1}{2}]dB(s)$$

where Γ is the standard Gamma function, H is the Hurst parameter on the interval $[0,1]$, t is time, and s is $t+\tau$ for a given time la g τ. The Hurst parameter describes the self-similarity of a given fBm process. A classical Brownian motion process with independent displacements has $H = 0.5$. $H > 0.5$ describes a process with positively correlated displacements, while $H < 0.5$ describes a process with negatively correlated displacements.

The Heteromotility software estimates the Hurst exponent based on Mandelbrot's Rescaled Range method, as described (Benoit B Mandelbrot and Wallis 1969). Briefly, for a given time series X of length N, the series is divided into a set of sub-series length $n = N/2^0, N/2^1, N/2^2, ..., N/2^\alpha$ where α is the largest power of 2 less than $\frac{1}{2}N$.

For each of these sub-series length n, the cumulative sum series Z is calculated on a mean adjusted time series $Y = X_t - \langle x \rangle$. A range $R(n) = max(Z) - min(Z)$ is calculated, along with the standard deviation $S(n) = stdev(X)$. The average rescaled range $R(n)/S(n)$ is calculated for each sub-series length n. The relationship between $R(n)/S(n)$, n, and H can be expressed generally as $E[R(n)/S(n)] = Cn^H$ where C is a constant coefficient. A simple log-log transformation is used to estimate H as the slope of a line $\log R(n)/S(n) = H \log Cn$.

Autocorrelation

Autocorrelation describes the correlation of a given process with itself across an interval of time. Expressed formally, autocorrelation R as a function of t for a given time lag τ:

$$R(t) = \frac{E[(X_t - \mu_t)(X_{t+\tau} - \mu_{t+\tau})]}{\sigma_t \sigma_{t+\tau}}$$

We estimate the autocorrelation for a cell's displacement series X of length T with a given time lag τ as:

$$R(\tau) = \frac{1}{(T-\tau)s^2}\sum_{t=1}^{T-\tau}(X_t - \langle X \rangle)(X_{t+\tau} - \langle X \rangle)$$

where s is the standard deviation of X.

Motion Model Simulations

Simulated data sets analyzed in this study were generated for a range of track lengths $T \in \{50, 100, 500\}$ with $n = 50000$ members per class. Data sets for analysis were resampled from these populations (with replacement) with sample sizes $n \in \{100, 500, 1000, 5000\}$ members per class, three times each per sample size/track length combination. Results for unsupervised clustering and supervised classification accuracy are reported as averages across all sampled populations for a given parameterization (i.e. sample size, track length).

Simulations were initially generated with identical mean displacement magnitudes ($d_{xy} = 5$) for each model type to prevent the trivial discrimination of different models. Simulations were also generated with varied displacement sizes, and different values of characteristic parameters to demonstrate robustness, as detailed below. These simulations were analyzed with a path length of $t = 100$ and sample size $n = 1000$.

Random Walks

We model an unbiased random walk is modeled as described (Berg 1993). For a walk of length T with step lengths t, a displacement is made in a random direction on the xy-plane for each time step. Displacement magnitudes are sampled from a Gaussian distribution with a given mean μ and variance $\sigma = \mu/5$. The mean displacement size is set to $d_{xy} = 5$ for initial experiments, and $d_{xy} \in \{10, 20, 50\}$ for experiments with varied parameters. Biased random walks are modeled in the same manner, but directionality is biased to step in a randomly chosen direction in the xy-plane $b\%$ of the time. The bias parameter is set to $b = 0.75$ for experiments with differing path lengths, and is varied to $b \in \{0.10, 0.70, 0.90\}$ for experiments with varied parameters.

Levy flight

Levy flights are modeled in the same manner as unbiased random walks, but displacement sizes are sampled from a Levy-like power law distribution $P(l_i) \propto l_j^{-\mu}$. We set the parameter $\mu = 2$ for intial experiments, as this models an optimized random search (Viswanathan et al. 1999). Experiments with varied parameters set $\mu \in \{0.5, 5\}$.

Fractal Brownian Motion (fBm)

A random walk is simulated as above with displacement sizes generated by a fractal Brownian motion process with a given Hurst coefficient $H = [0, 1]$. This fBm process is simulated by the Cholesky decomposition method, as outlined (Dieker 2004). Briefly, a matrix $\Gamma = (R(t_i, t_j), i, j)$ is generated for $i, j = 0...n$ where $R(t, s) = (s^{2H} + t^{2H} - |t - s|^{2H})/2$. A matrix $\Sigma = \Gamma^{\frac{1}{2}}$ is computed as the lower triangle L returned by Cholesky decomposition of Γ. A vector v of length n containing normally distributed values is generated. The vector $u = v\Sigma$ represents a series of fBm displacements. For the simulation analysis in this paper, the Hurst parameter was set to $H = 0.9$ to generate a displacement series with long-range memory in initial experiments. Experiments with varied parameters set $H \in \{0.1, 0.5, 0.99\}$.

Unsupervised Clustering

Hierarchical Clustering

All data were scaled $[-1, 1]$ with unit variance prior to cluster analysis. Hierarchical clustering was performed with Ward's linkage using the standard R stats library. The appropriate number of clusters k for each system was determined using a panel of 30 cluster validation indices (implemented by the NbClust R package (Charrad et al. 2014)), considering the relative performance of different indices (Arbelaitz et al. 2013). These indices were determined using the first thirty principal components for each system as features to avoid colinearity while retaining >=95% of total variation. The biological relevance of cluster phenotypes for each k was also considered. All clustering partitions utilized demonstrate a positive Silhouette index, providing evidence of an appropriate cluster structure (Rousseeuw 1987). Beyond optimization of the Silhouette value, we consider the TraceW, Marriot, and D-index with higher weight when a clear consensus vote of all indices is not present. The TraceW and Marriot indices perform well on problems with uneven cluster size when compared to others (Dimitriadou, Dolničar, and Weingessel 2002), and the D-index allows for graphical interpretation of partition validity (Charrad et al. 2014).

When a consensus vote is non-obvious, we also perform clustering on subsamples of the data set with candidate cluster partition numbers k to evaluate the phenotypic stability of clusters. Subsampling is performed by drawing a random 80% of cells in a population for each sample. We present such an analysis for the MuSC system in (S13 Fig), demonstrating that a 3 cluster partition yields stable cluster phenotypic definitions while a 4 cluster partition does not.

Unsupervised clustering accuracy for simulated data was determined as the percentage of samples that reside in a cluster where that sample's type (i.e. Random walk, Levy flier) is the majority sample type for the cluster. For instance, a Random walker simulation that lies in a cluster with a majority of other Random walker samples is considered an accurate clustering, while a Power flier that lies in a cluster with majority Random walkers is considered an incorrect clustering.

t-Stochastic Neighbor Embedding

t-Stochastic Neighbor Embedding was performed as described (L van der Maaten 2014) using the *Rtsne* implementation (Krijthe 2015). The perplexity parameter of t-SNE was chosen by computing the t-SNE projection at a series of perplexity values in a range from 10 to 70 and evolving for 5000 iterations, as suggested by the algorithm's authors (Laurens van der Maaten and Hinton 2008). Between perplexity values of 30 and 70, we find that the global structure of t-SNE projections is relatively consistent. From this range, we choose the value that provides the most consistent projections for the data set, as suggested in recent work on the proper use of t-SNE (Wattenberg, Viégas, and Johnson 2016).

Independent Component Analysis

Independent component analysis was performed as described (Hyvarinen and Oja 2000) using the *ica* R package (Helwig 2015).

Pseudotiming

Pseudotiming was performed using the Monocle package (Trapnell et al. 2014) on all myoblast samples and a subset of ~800 randomly selected MuSC cells (MuSC data was subsampled to reduce computational expense). Monocle's Discriminative Dimensionality Reduction with Trees algorithm was used for dimensionality reduction, and the top 20 features loaded into the first principal component were used in place of "ordering genes."

Statistical analysis

MANOVAs comparing all feature means between clusters were performed using the first 30 principle components as measured variables for each cell. t-tests were performed on unscaled feature values, assuming a two-sided null hypothesis and unequal sample variance. One-way ANOVAs comparing feature means between clusters were performed on unscaled feature values. The binomial test was performed on pairwise transitions to determine if transitions were non-symmetrical using the $H_0 : P(success) = 0.5$. All tests were performed with R standard library and Python "statsmodels" implementations.

Local Cell Density Analysis

The cell density for each cell was represented using a "Local Cell Density Index" computed as the sum of inverse squares of distances to neighboring cells, as

$$LCD_i = \sum_{j=1}^{N} d(i,j)^{-2}$$

where LCD_i is the Local Cell Density Index for cell i, j is the index of each other cell in the field-of-view, N is the total number of other cells in the field-of-view, and d is the Euclidean distance function. The Local Cell Density Index is therefore higher when many neighboring cells are nearby, and lower when there are few nearby neighbors. We justify scaling the contribution of neighboring cells by the inverse squared distance based on the properties of diffusible signals (Berg 1993).

Notably, only cells that were tracked in a given field-of-view can be evaluated as neighbors. As our current tracking paradigm does not track every cell, the Local Cell Density Index we calculate is an imperfect estimation of the true local cell density.

Relationships between each Heteromotility feature analyzed and the Local Cell Density Index were evaluated by fitting linear regression models of the form

$$LCD = m(Feature_i) + b$$

for all features i in the set.

The strength of the relationship is assessed based on Pearson's r^2 value for the regression and the statistical significance is evaluated based on the F-test.

Analysis of State Transition Dynamics

Subpath Analysis

To capture motility features at multiple time points for state transition analysis, cell paths are segmented into various subpaths of length τ and features extracted from each subpath. Analysis presented in the manuscript are performed with $\tau \in \{20, 25, 30\}$, as indicated. Heteromotility contains features to perform this segmentation and analysis of subpaths, accessible via a command line flag. There is a lower boundary of $\tau = 20$ for subpath size, as multiple features calculated by Heteromotility require at least 20 time points for analysis.

State Specific State Transition Quantification

State transitions within each state cluster, as presented in (Fig. 4C) are quantified as follows. Cluster identities are assigned using hierarchical clustering of the full-length motility track, as noted above. Full length tracks are then transformed into PCA space. Paths are segmented into $\tau = 20$ length subpaths, and Heteromotility features were extracted from each subpath. Subpaths are then transformed into the PCA space defined for full length tracks, such that the principal components for full length tracks are the same for subpaths. Transitions for each cell are quantified as the vector V between each pair sequential subpaths $S_0 \rightarrow S_1$ along the first two principal components (PCs).

The mean transition vector V_c for a cluster $c \in C$, where C is the set of all clusters is defined then as:

$$V_c = \frac{\sum_{i=1}^{N} \sum_{t=1}^{T-1} S_i(t+1) - S_i(t)}{N(T-1)}$$

where N is the number of cells in cluster c, T is the maximum time interval considered, and $S_i(t)$ is the state position in principal component space of cell i at time interval t.

The mean of all transition vectors for all cells with a given cluster identity is taken as the mean transition vector for a given cluster. These vectors are plotted atop a PCA projection of the full-length path state space, with an origin at the cluster centroid. Vectors in plots are presented with 10X magnitude to enhance visualization. Transition vector magnitudes are calculated as the magnitude of the mean vector, plus or minus the standard error of the mean (SEM) pooled across dimensions ($SE_{xy} = \sqrt{SE_x^2 + SE_y^2}$).

Coarse-grained Probability Flux Analysis (cgPFA)

Coarse-grained probability flux analysis (cgPFA) as presented in (Fig. 5) is implemented per the definition of Battle *et. al.* (Battle et al. 2016). Briefly, for each system, PCA is performed on the Heteromotility feature set extracted from subpaths under consideration and the first N principal components are used to define a motility state α. Each principal component is "coarse-grained" by binning into k subsections of equal length. Combinations of bins between each of the N coarse-grained principal components define a given state α. coarse-grained PFA analysis presented here was performed for a set of dimensionality resolutions N, where $N \in \{1, 2, 3, 4\}$ and a set of coarse-grained resolutions k for each dimensionality resolution N, where $k \in \{2, 3, 5, 7, 10, 15, 20\}$. Explicitly, cgPFA was performed in 1D, 2D, 3D, and 4D, and detailed balance was evaluated with binning schemes using 2, 3, 5, 7, 10, 15, or 20 bins. Overlapping window experiments were performed by setting the stride for τ length subpaths at a value of stride $s < \tau$. In all overlapping window experiments, three windows were considered. Code for all our coarse-grained PFA implementations described is available in the Heteromotility GitHub repository https://github.com/cellgeometry/heteromotility.

Two-dimensional cgPFA state space representations

To produce 2D state vector plots as presented in (Fig. 5), each cell's state, per the above definition, is considered for the each subpath t_i, and the subsequent subpath $t_i + 1$ for $i \in [1, T - 1]$. Each cell's state transition is recorded as a two-dimensional vector $v = (x_{PC1}, y_{PC2})$ representing the distance in $N = 2$ dimensions of PC space the cell traveled in that time interval. If the cell did not travel to a directly neighboring state (a unit vector displacement), the cell's path is interpolated as a series of unit vectors between the initial and final state. Each of these interpolated displacements is recorded in an intermediary bin, representing the assumption that cells traverse state space in a linear fashion.

For each state α, the transition rate from that state is calculated as the vector mean of all transition vectors originating in that state. Non-transitioning cells (zero magnitude transition vectors) are not considered in calculating this transition rate. These transition rates are plotted atop the binned state space as arrows, such that the magnitude of each arrow represents the magnitude of the transition rate constant, and arrow direction the direction of the transition rate constant in a given bin. The divergence of this vector field is calculated and presented as a heatmap on the state space. The divergence at a given state serves as a metric of state stability. Metastable states display negative divergence (more cells entering than exiting), while unstable states display positive divergence (more cells exiting than entering).

Detailed Balance assessment by N-dimensional cgPFA

To provide statistical assessment of detailed balance as presented in (Fig. 6), a state space is represented in N dimensions using the first N principal components, and each of these N dimensions is coarse-grained into k equally sized bins, as described above. A matrix M of dimensionality $2N$ is generated with k bins in each dimension is generated, representing all possible state transition combinations in state space. The first N dimensions of the space represent a cell's initial state in the space, and the next N dimensions represent the final or destination state across a time interval. To record a state transition, the value in the corresponding bin is iterated by 1.

For example, in a $N = 3$ dimensional space coarse-grained with $k = 3$, a 6 dimensional matrix is generated with $k = 3$ bins per dimension, for a total of $k^N = 3^6$ bins. A transition from a cell that begins at location

$(1, 4, 3)$ and ends at location $(2, 4, 2)$ in state space would be recorded by iterating the value of $M(1, 4, 3, 2, 4, 2)$ by 1. This process is repeated for each cell in the population, and each transition for each cell.

A state space in perfect detailed balance would be expected to have all pairwise transition rates $A \rightarrow B = B \rightarrow A$. In the matrix M, this would manifest as equal values in all pairwise sets of bins $M(a, b, c, d, e, f) = M(d, e, f, a, b, c)$ (for a $N = 6$ dimensional case). We test detailed balance by checking the pairwise balance of all such state transition sets using the binomial test with $H_0 : p = 0.5$ where p is the probability of cells falling into either bin. If this null hypothesis H_0 is rejected, it indicates that a pairwise transition set is unbalanced, and therefore that detailed balance is broken in the system. All binomial tests are p-value corrected with $\alpha = 0.05$ using the Benjamini-Hochberg method to account for multiple hypothesis testing.

For each system presented, we test detailed balance breaking in this manner for $N \in \{1, 2, 3, 4\}$ and $k \in \{2, 3, 5, 7, 10, 15, 20\}$. As $N > 2$ dimensional spaces are challenging to present in their entirety, we display the top 5 most unbalanced (lowest binomial test p values) as a heatmap, with the initial state in the left column and the destination state in the right column. We note that these tests are biased toward Type II error, as linear binning in N dimensions may not accurately capture true states and binomial tests are underpowered when testing rare state transitions.

Dwell Time Quantification

Characteristic dwell times for each state were quantified in the N dimensional course-grained PCA spaces described above. For each dimensional resolution N and course-grained resolution k, each state S_i is considered. States S_i with fewer than 10 observed dwells are omitted to avoid inaccurate time constant approximation. The dwell time in discrete time units for each cell observed in a state S_i during the series was recorded. For instance, if a cell is observed in state S_i at time points $t = 1$ and $t = 2$, then leaves the state on $t = 3$, a dwell time of two time units is recorded. If a cell has multiple dwells in state S_i, separated by a dwell in another state, multiple dwell times of observed length are recorded. The result of this procedure is a table of observed dwells for each possible discrete time length $t \in [1, T]$ where T is the total length of the observation period in discrete time units. The dwell time for each state is characterized by two means: mean estimates and fitting of exponential decay functions to estimate time constants. Dwell times in Markovian systems are exponentially distributed. Simple arithmetic means are not an appropriate maximum likelihood estimate (MLE) for exponentially distributed data, and as such alternative MLE methods and exponential decay curve fitting are employed (Milescu et al. 2006; Landowne, Yuan, and Magleby 2013). We apply the latter approach and fit exponential decay functions to the number of cells that would be observed at each discrete time point (effectively, the cumulative sum of dwell times, such that all cells are observed at $t = 1$, cells dwelling $t = 2$ units or longer are observed at $t = 2$, and so on). Curves are of the form

$$y(t) = a \exp(\frac{-t}{\tau})$$

where τ is the time constant of decay, a is a parameter coefficient, and C is a parameter constant and are fit using a differential evolution method, similar to that described in (Zou and Larson 2014). Distribution of discrete dwell time data was assessed graphically relative to a binned values from a theoretical exponential distribution with the shape parameter $\lambda = \bar{X}$, where $\bar{X}$ is the mean of the sample observation distribution X (Lilliefors 1969).

Hierarchical Clustering PFA

Probability flux analysis was also performed using hierarchical clustering (Fig. S6) to define cell state for a given time interval, in contrast to the use of coarse-grained principal components as above. To statistically assess if detailed balance is broken using hierarchical cluster-based stated definitions, we represent state transitions as an N-by-N matrix, where N is the number of hierarchical clusters in the partitioning scheme. Rows of the matrix represent a given cell's state at the an initial time interval t_0, while columns represent the cell's state at the subsequent time interval t_1. Values in each i, j-indexed cell of the matrix represent the

number of cells meeting the described state criteria (state i at t_0, state j at t_1). As in statistical assessment of one dimensional coarse-grained PFA, a system in detailed balance would display symmetry about the diagonal, and symmetrical bins about the diagonal represent pairwise state flux magnitudes. Symmetry breaking is statistically tested using a binomial test for cells falling above or below the diagonal. The same Type II error caveat applies, as this approach only tests for raw numbers of cells above and below the diagonal, and does not test for proper pairwise transition symmetry. Therefore, statistical testing of detailed balance breaking by hclust-PFA is biased toward *false negative* conclusions, rather than *false positives*. This statistical testing approach is generalizable to any arbitrary state definition scheme. We also test the equivalency of pairwise transition rates for detailed balance by the binomial test, as described for N-dimensional coarse-grained PFA above. Code for our hierarchical clustering PFA implementation is available in the Heteromotility GitHub repository.

Supervised Machine Learning Classification

Machine learning models utilized standard scikit-learn (Pedregosa et al. 2011) and R implementations. All data were scaled $[-1, 1]$ with unit variance prior to model training.

Simulated Data Classification

For simulated data, Random Forests were instantiated with $n = 100$ estimators and R `randomForest` default parameters. Random Forest feature importance was determined as the decrease in classification accuracy when a feature was removed (Breiman 2001). Model accuracy was estimated by performing five-fold stratified, shuffled cross-validation. Confusion matrices are the mean predictions of a five-fold stratified split.

MEF Transformation State Classification

Classification of MycRas MEFs vs WT MEFs was performed using a Support Vector Machine. Support vector machines were chosen as the classification model based on a test of multiple model types using a simple 5-fold cross-validation scheme, in which SVMs performed best.

Classification was carried out in a "Round Robin" fashion across independent biological experiments. For the Round Robin, we train a classifier on 4 out of 6 independent experiments, and classify the remaining 2. The accuracy of classifying these 2 "held out" experiments is taken as the classification accuracy for that split. The process of training on 4 experiments, and classifying the remaining 2 is repeated for all possible training/testing combinations. The average classification accuracy across all splits is taken as the Round Robin Classification Accuracy. This Round Robin analysis represents a form of strong cross-validation, accounting for differences that may arise between individual biological experiments.

SVM parameters were optimized by a grid search using the Round Robin Classification Accuracy as the objective. Grid Search identified a radial basis function kernel, feature selection to 62% of features with top ANOVA F-values, and a regularization constant $C = 0.5$ as the optimal configuration. All classifier code is available in the Heteromotility Github repository (http://github.com/cellgeometry/heteromotility).

Heteromotility User Guide

For users interested in applying the Heteromotility tool to their own research problems, we have outlined recommendations for experimental design and parameter selection.

Timelapse Imaging Experimental Design

It is critical that timelapse imaging experiments be designed with sufficient temporal resolution to capture motility behaviors of interest. For instance, if a user is interested in motility processes that occur on the order of minutes, imaging should be performed on a higher sub-minute temporal resolution. Alternatively, if a user is interested in processes that take place on an hours or days long timescale, temporal resolution can be decreased to the order of minutes or fractions of hours. In the latter scenario, the total length of imaging should be designed to capture the whole process of interest.

Sample Size Determination

Depending on the downstream analysis desired, the number of cells that need to be analyzed varies. For supervised classification problems, we recommend users perform a multivariate power analysis. For unsupervised clustering, rigorous analytical tools to determine the necessary sample sizes are lacking. Due to the Curse of Dimensionality (Domingos 2012) principle, the number of samples necessary to define clusters increases with the dimensionality of the problem. We find in three distinct biological systems that 30 principal components are sufficient to capture the overwhelming majority of variation, and therefore we suggest it is reasonable for users to assume a $<= 30$ feature dimensions will be present in their unsupervised clustering problem. As a simple rule of thumb, we recommend users have at least 5 fold as many samples as features, such that at least 150 cells should be analyzed as a lower bound for unsupervised clustering. Again, we reiterate that the sample sizes necessary to elucidate structure through unsupervised clustering cannot be readily predicted *a priori*, and therefore we provide this sample size suggestion only as a lower bound for experimental planning.

Supervised Classification

Users interested in supervised classification of motility phenotypes will need to select a data preprocessing method and a classification model to train. As a starting point, we recommend scaling raw Heteromotility feature outputs on the range [-1, 1] and reducing dimensionality using principal component analysis (PCA) prior to training a classification model. We recommend users start with an ensemble classification method such as Random Forest Classifiers, as they are robust in a variety of classification problems (Caruana and Niculescu-Mizil 2006). For validation of efficacy, we recommend users perform at least five-fold cross-validation (five independent models, each trained on 80% of the data and tested on the remaining 20% in a rotating fashion). If multiple biological experiments are pooled for analysis, a round-robin analysis training on one set of experiments, and predicting on an independent set of experiments, is also useful to assess the robustness of the trained classification model. We recommend users perform a Grid Search to select hyperparameters for a given classifier. A practical guide to grid search implementation can be found in (Hsu, Chang, and Lin 2003).

Unsupervised Clustering

For unsupervised clustering of motility feature data, we recommend users begin by scaling raw Heteromotility features [-1, 1]. Dimensionality should be reduced using PCA prior to clustering. We recommend users retain the smallest number of PCs sufficient to explain ~90% of variation. Clustering may be performed using hierarchical clustering. We recommend using Ward's linkage, as this yields the most intuitive results in our systems and makes assumptions about cluster shape in line with biological priors. The number of clusters k to be defined may be optimized using cluster validity indices. We recommend the R package "NbClust" to compute several of these indices. While there is no definitive index that dominates others in all contexts, we recommend that users prioritize optimization of the Silhouette value, TraceW index, Marriot index, and D index second derivative. These metrics are perform well on unbalanced clustering problems, and the D-index provides a graphical interpretation (Arbelaitz et al. 2013; Dimitriadou, Dolničar, and Weingessel 2002). We also recommend users perform a resampling analysis to test the robustness of their cluster partition to sample

fluctuations. We perform these analysis by sampling 80% of cells in a population and applying a candidate cluster partitioning scheme over several iterations. Partitions which produce clusters with similar phenotypic character across sample draws may be considered more robust.

Feature Dimensionality

For both Supervised and Unsupervised analysis of Heteromotility feature data, the dimensionality of the feature space effects the efficacy of analysis. The "Curse of Dimensionality" (Steinbach, Ertoz, and Kumar 2004) reduces the efficacy of supervised learning and unsupervised clustering when many low-information dimensions are present. To avoid this problem, we recommend users reduce the dimensionality of input spaces using PCA prior to analysis, as outlined above. Users may wish to further reduce dimensionality by eliminating features prior to PCA, or using fewer principal components if they believe a Curse of Dimensionality issue is present. If a user wishes to combine Heteromotility features with other phenotypic features, the user should be mindful of the Curse of Dimensionality and take steps to reduce the dimensionality of the feature space where possible. However, the removal of features pre-maturely may hinder analysis that relies on a discarded feature for discrimination of two samples. Therefore, we recommend users attempt analysis with PCs covering a large proportion of the population variation first, before discarding additional features.

t-SNE non-Linear Dimensionality Reduction Visualization

For presentation of Heteromotility feature space, we recommend the use of t-Stochastic Neighbor Embedding (t-SNE) (Laurens van der Maaten and Hinton 2008). When utilizing t-SNE, it is critical that users understand the limitation on interpretation of t-SNE visualizations, as recently described in (Wattenberg, Viégas, and Johnson 2016). The critical hyperparameter in t-SNE analysis is perplexity. As outlined by the algorithm's authors (Laurens van der Maaten and Hinton 2008), appropriate values of perplexity cannot be determined *a priori*, and vary depending on the data set. In general, larger data sets with more data points will benefit from a higher setting of perplexity. We recommend users generate multiple maps for several settings of perplexity in the range [10, 70], selecting the a perplexity value that generates qualitatively reproducible representations.

Motility State Transition Quantification

In quantifying motility state transitions, the time interval hyperparameter for state definition τ must be chosen by the user. This value represents the number of time steps considered in each temporal window where the motility state of a cell is defined. Several Heteromotility features lose numerical stability if applied to series shorter than $\tau = 20$ time steps. A setting of $\tau = 20$ therefore represents a lower bound for the time scale of analysis. We recommend users utilize this lower bound parameter value in most cases, as this will allow for measurement of all transitions detectable given the temporal resolution of the experiment.

An exception to this suggestion is if a user is interested in the potential for detailed balance breaking on time scales of a longer order than a $\tau = 20$ window. This may occur for example if a user is interested in determining whether phenotypic transitions among a neoplastic population that take place over days are in detailed balance, but images every 30 seconds such that $\tau = 20$ windows are only 10 minutes of real time. In this instance, we suggest the user select a value of τ on the same order as the state durations they wish to measure. This avoids detection of temporary detailed balance breaking on time scales the user is uninterested in. This could occur in our example above if cells in one phenotypic state display an intrastate flux loop through substates of motility behavior. In this instance, sampling at $\tau = 20$, 10 minute intervals may detect detailed balance breaking on this timescale, even if it is not present on the days long timescale of interest, confounding results.

Additional Supplemental Videos

For additional supplemental videos, please visit our lab website at http://cellgeometry.ucsf.edu/heteromotility.

References

Arbelaitz, Olatz, Ibai Gurrutxaga, Javier Muguerza, Jesús M Pérez, and Iñigo Perona. 2013. "An extensive comparative study of cluster validity indices." *Pattern Recognition.*

Battle, Christopher, Chase P Broedersz, Nikta Fakhri, Veikko F Geyer, Jonathon Howard, Christoph F Schmidt, and Fred C MacKintosh. 2016. "Broken detailed balance at mesoscopic scales in active biological systems." *Science (New York, N.y.).*

Berg, Howard C. 1993. *Random Walks in Biology.* Princeton University Press.

Breiman, L. 2001. "Random forests." *Machine Learning.*

Caruana, Rich, and Alexandru Niculescu-Mizil. 2006. "An Empirical Comparison of Supervised Learning Algorithms." In *International Conference on Machine Learning.*

Chakkalakal, Joe V, Josef Christensen, Wanyi Xiang, Mathew T Tierney, Francesca S Boscolo, Alessandra Sacco, and Andrew S Brack. 2014. "Early forming label-retaining muscle stem cells require p27kip1 for maintenance of the primitive state." *Development.*

Charrad, M, N Ghazzali, V Boiteau, and A Niknafs. 2014. "NbClust package for determining the number of clusters in a dataset." *Journal of Statistical Software.*

Dieker, T. 2004. "Simulation of fractional Brownian motion." PhD thesis.

Dimitriadou, Evgenia, Sara Dolničar, and Andreas Weingessel. 2002. "An examination of indexes for determining the number of clusters in binary data sets." *Psychometrika.*

Domingos, Pedro. 2012. "A Few Useful Things to Know About Machine Learning." *Communications of the Acm.*

E Michalska, Anna. 2007. "Isolation and propagation of mouse embryonic fibroblasts and preparation of mouse embryonic feeder layer cells." *Current Protocols in Stem Cell Biology.*

Helwig, Nathaniel E. 2015. "Independent Component Analysis."

Hsu, Chih-Wei, Chih-Chung Chang, and Chih-Jen Lin. 2003. "A Practical Guide to Support Vector Classification." *NTU.*

Hyvarinen, A, and E Oja. 2000. "Independent component analysis: algorithms and applications." *Neural Networks.*

"*Image Analysis and Mathematical Morphology.*" n.d.

Jacob, M, and M Unser. 2004. "Design of steerable filters for feature detection using Canny-like criteria." *IEEE Transactions on Pattern Analysis and Machine Intelligence.*

Jaqaman, Khuloud, Dinah Loerke, Marcel Mettlen, Hirotaka Kuwata, Sergio Grinstein, Sandra L Schmid, and Gaudenz Danuser. 2008. "Robust single-particle tracking in live-cell time-lapse sequences." *Nature Methods.*

Krijthe, J. 2015. *Rtsne: T-Distributed Stochastic Neighbor Embedding using Barnes-Hut Implementation (R package version 0.10).* Computer Software.

Landowne, David, Bin Yuan, and Karl L Magleby. 2013. "Exponential Sum-Fitting of Dwell-Time Distributions without Specifying Starting Parameters." *Biophysj.*

Lilliefors, H W. 1969. "On the Kolmogorov-Smirnov test for the exponential distribution with mean unknown."

Journal of the American Statistical Association.

Maaten, L van der. 2014. "Accelerating t-SNE using tree-based algorithms." *Journal of Machine Learning Research.*

Maaten, Laurens van der, and Geoffrey Hinton. 2008. "Visualizing Data using t-SNE." *Journal of Machine Learning Research.*

Mandelbrot, B B, and J W Van Ness. 1968. "Fractional Brownian motions, fractional noises and applications." *SIAM Review.*

Mandelbrot, Benoit B, and James R Wallis. 1969. "Robustness of the rescaled range R/S in the measurement of noncyclic long run statistical dependence." *Water Resources Research.*

Milescu, Lorin S, Ahmet Yildiz, Paul R Selvin, and Frederick Sachs. 2006. "Maximum likelihood estimation of molecular motor kinetics from staircase dwell-time sequences." *Biophysj.*

Otsu, N. 1975. "A threshold selection method from gray-level histograms." *Automatica.*

Pedregosa, Fabian, Gaël Varoquaux, Alexandre Gramfort, Vincent Michel, Bertrand Thirion, Olivier Grisel, Mathieu Blondel, et al. 2011. "Scikit-learn: Machine Learning in Python." *Journal of Machine Learning Research.*

Rahman, A. 1964. "Correlations in the Motion of Atoms in Liquid Argon." *Physical Review.*

Rousseeuw, P J. 1987. "Silhouettes - a Graphical Aid to the Interpretation and Validation of Cluster-Analysis." *Journal of Computational and Applied Mathematics.*

Shea, Kelly L, Wanyi Xiang, Vincent S LaPorta, Jonathan D Licht, Charles Keller, M Albert Basson, and Andrew S Brack. 2010. "Sprouty1 regulates reversible quiescence of a self-renewing adult muscle stem cell pool during regeneration." *Cell Stem Cell.*

Steinbach, Michael, Levent Ertoz, and Vipin Kumar. 2004. "New Directions in Statistical Physics." In *The Challenges of Clustering High Dimensional Data.*

Trapnell, Cole, Davide Cacchiarelli, Jonna Grimsby, Prapti Pokharel, Shuqiang Li, Michael Morse, Niall J Lennon, Kenneth J Livak, Tarjei S Mikkelsen, and John L Rinn. 2014. "The dynamics and regulators of cell fate decisions are revealed by pseudotemporal ordering of single cells." *Nature Biotechnology.*

Truitt, Morgan L, Crystal S Conn, Zhen Shi, Xiaming Pang, Taku Tokuyasu, Alison M Coady, Youngho Seo, Maria Barna, and Davide Ruggero. 2015. "Differential Requirements for eIF4E Dose in Normal Development and Cancer." *Cell.*

Viswanathan, G M, S V Buldyrev, S Havlin, M G da Luz, E P Raposo, and H E Stanley. 1999. "Optimizing the success of random searches." *Nature.*

Wattenberg, Martin, Fernanda Viégas, and Ian Johnson. 2016. "How to Use t-SNE Effectively." *Distill.*

Zou, Weizhong, and Ronald G Larson. 2014. "A mesoscopic simulation method for predicting the rheology of semi-dilute wormlike micellar solutions." *Journal of Rheology.*
