# Supplementary figures and images for: Inferring cell state by quantitative motility analysis reveals a dynamic state system and broken detailed balance
Source: PLoS Comput Biol. 2018 Jan 16;14(1):e1005927. doi: 10.1371/journal.pcbi.1005927 (PMC5786322; doi:10.1371/journal.pcbi.1005927)

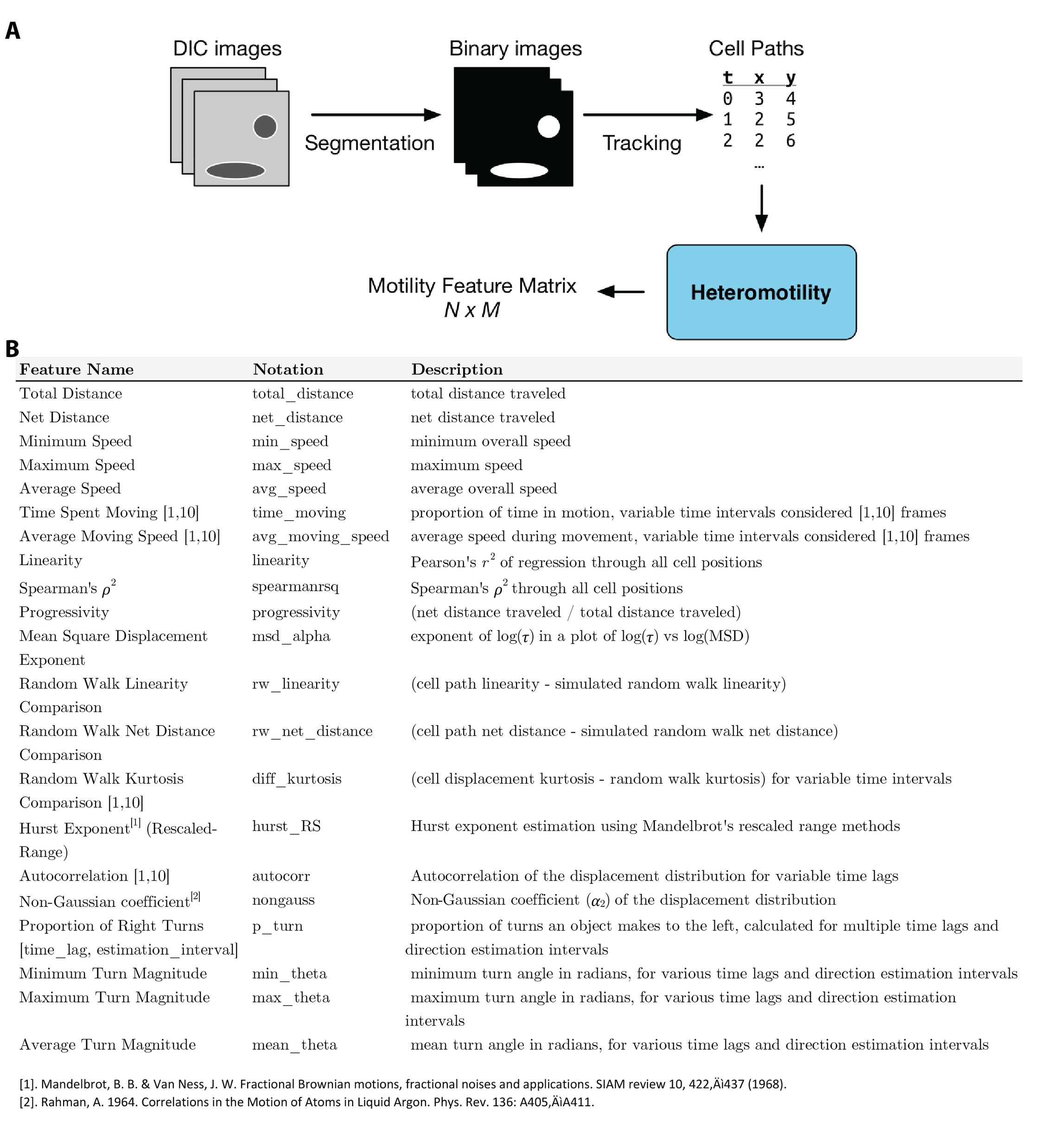

Supplement: S1 Fig — (A) Heteromotility workflow diagram and (B) a table of the complete feature set. (TIF) [file pcbi.1005927.s002.tif]

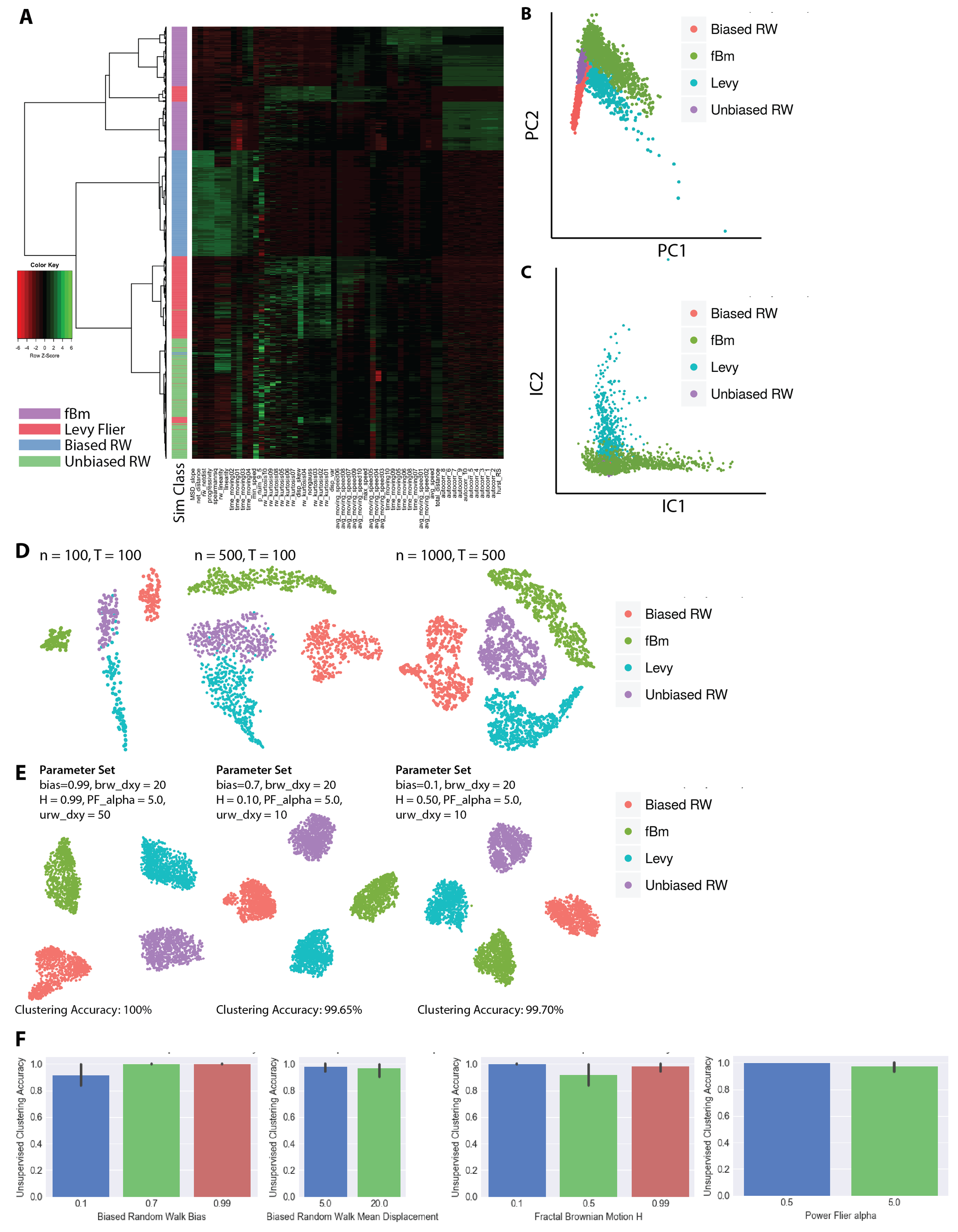

Supplement: S2 Fig — (A) Simulated models of motion are segregated by unsupervised heirarchical clustering, as displayed in representative a heatmap of hierarchically clustered (Ward’s linkage) simulated motion paths (1000 members/class, length 100 time units) based on Heteromotility features. Color labels on the left mark a sample’s True Class. Effective separation of the True Classes indicates effective detection of different phenotypes by unsupervised clustering. Two-dimensional (B) PCA and (C) ICA visualization of the simulated motion paths to provide an intuition for the linearity of motility state space and performance of traditional linear dimensionality reduction techniques. Only high (30) dimensional PCA spaces are used for analysis. ICA is not used for any downstream analysis. (D) Representative t-SNE visualizations of simulated motion models with different sample sizes and track lengths, labeled with ground truth classes. Models occupy distinct regions of state space under all sample size and track length variations. (E) Representative t-SNE visualizations of simulated motion model groups with the underlying parameters for each motion model varied. Parameters for each condition shown are displayed above the t-SNE map. (F) Unsupervised clustering accuracy (Ward’s linkage) as a function of parameter variations to the underlying simulations. Performance decreases as expected when parameters are set in a manner that decreases the distinctness of the models. For example, performance is lower when the bias parameter for biased random walks is set to a low value, close to an unbiased random walk, or when the fractal Brownian motion index is set to the same index displayed by a random walker (H = 0.5). Performance is high across other conditions tested. (TIF) [file pcbi.1005927.s003.tif]

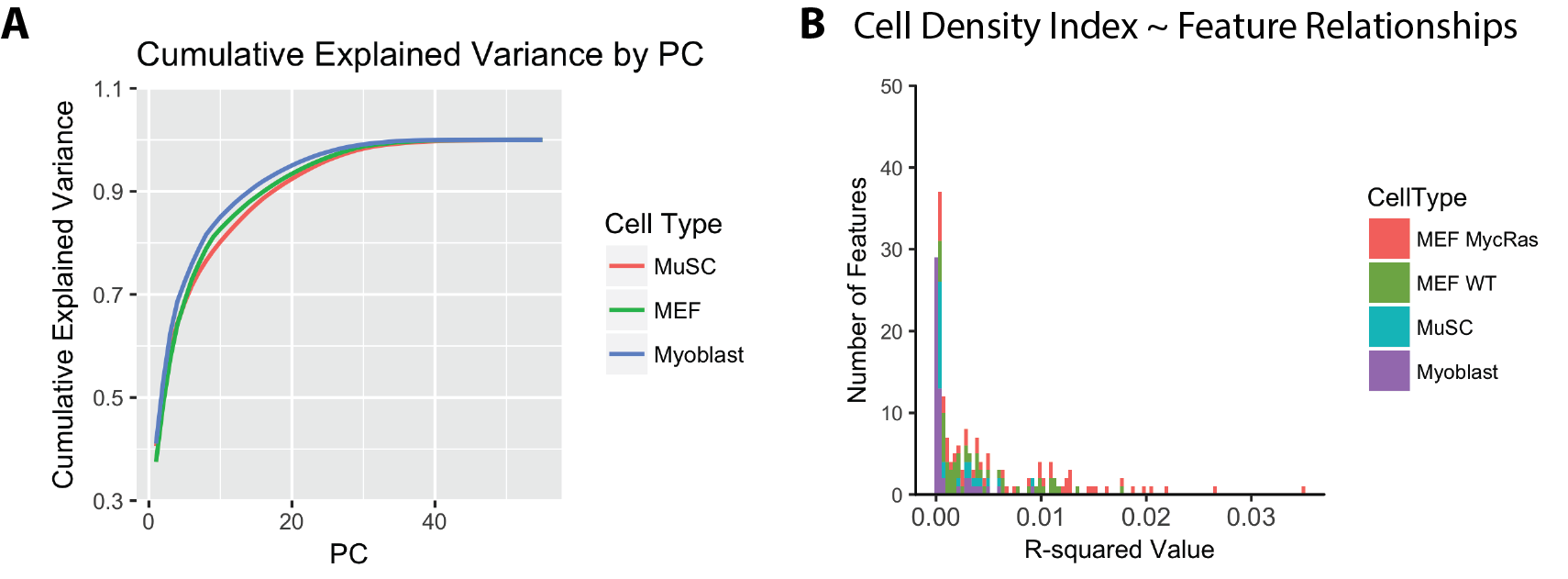

Supplement: S3 Fig — (A) Cumulative variance explained for each dimensionality of principal component space across MuSC, MEF, and Myoblast systems. (B) Strength of relationships between our Local Cell Density Index and each of the Heteromotility features, displayed as overlapping histograms of Pearson’s r2 values for linear regression models fit between the Local Cell Density Index and each feature pairwise. No features in any system display a relationship with a meaningful effect size (max r2 ≈ 0.03). (TIF) [file pcbi.1005927.s004.tif]

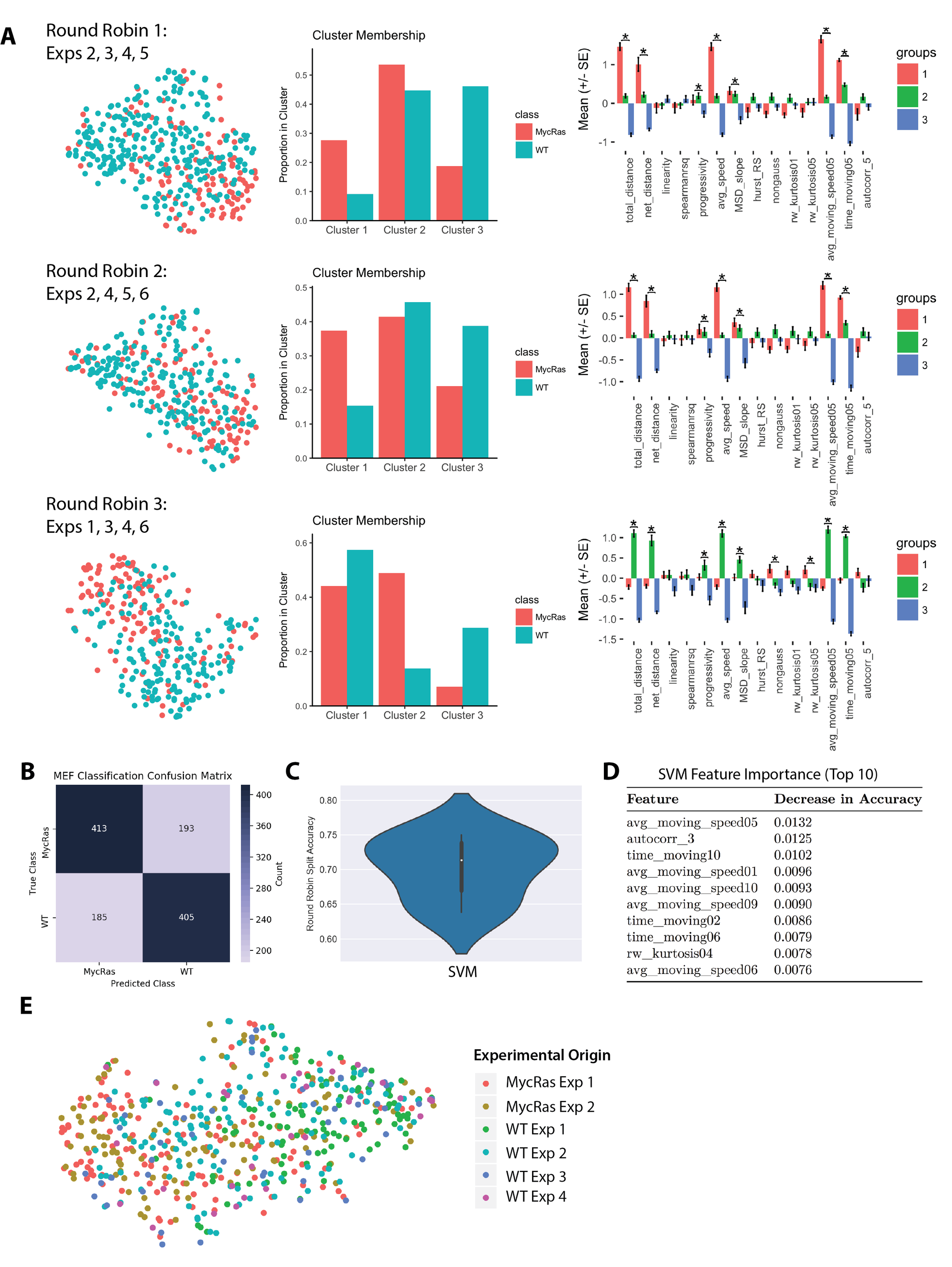

Supplement: S4 Fig — (A) Representative t-SNE visualizations, MycRas and wild-type cluster distrobutions, and selected cluster feature values for three “splits” from a Round Robin analysis. In the Round Robin analysis, analyses were performed on 4 experiments (1 MycRas, 3 wild-type) leaving 2 experiments out in an iterative fashion for each possible combination of experiments. MEF cluster distributions and properties are reproducible across all combinations. (B) Aggregate confusion matrix for SVM classifiers trained in a Round Robin fashion, such that 4 experiments were used for training and 2 for evaluation for all combinations of 4 experiments. SVMs classify MycRas and wild-type cells with 70% accuracy and do not demonstrate a prediction bias for one class over the other. (C) Distribution of Round Robin Classification Accuracies for SVMs trained on each Round Robin split. (D) Top 10 most important features for Round Robin Classification Accuracy using SVM classifiers. Features importance is determined as the decrease in classification accuracy when an SVM is retrained without a given feature as input. The Top 10 features were selected based on the mean decrease in Round Robin Classification Accuracy across Round Robin splits. (E) t-SNE visualization of all MEF cells analyzed, labeled with their experimental origin. (TIF) [file pcbi.1005927.s005.tif]

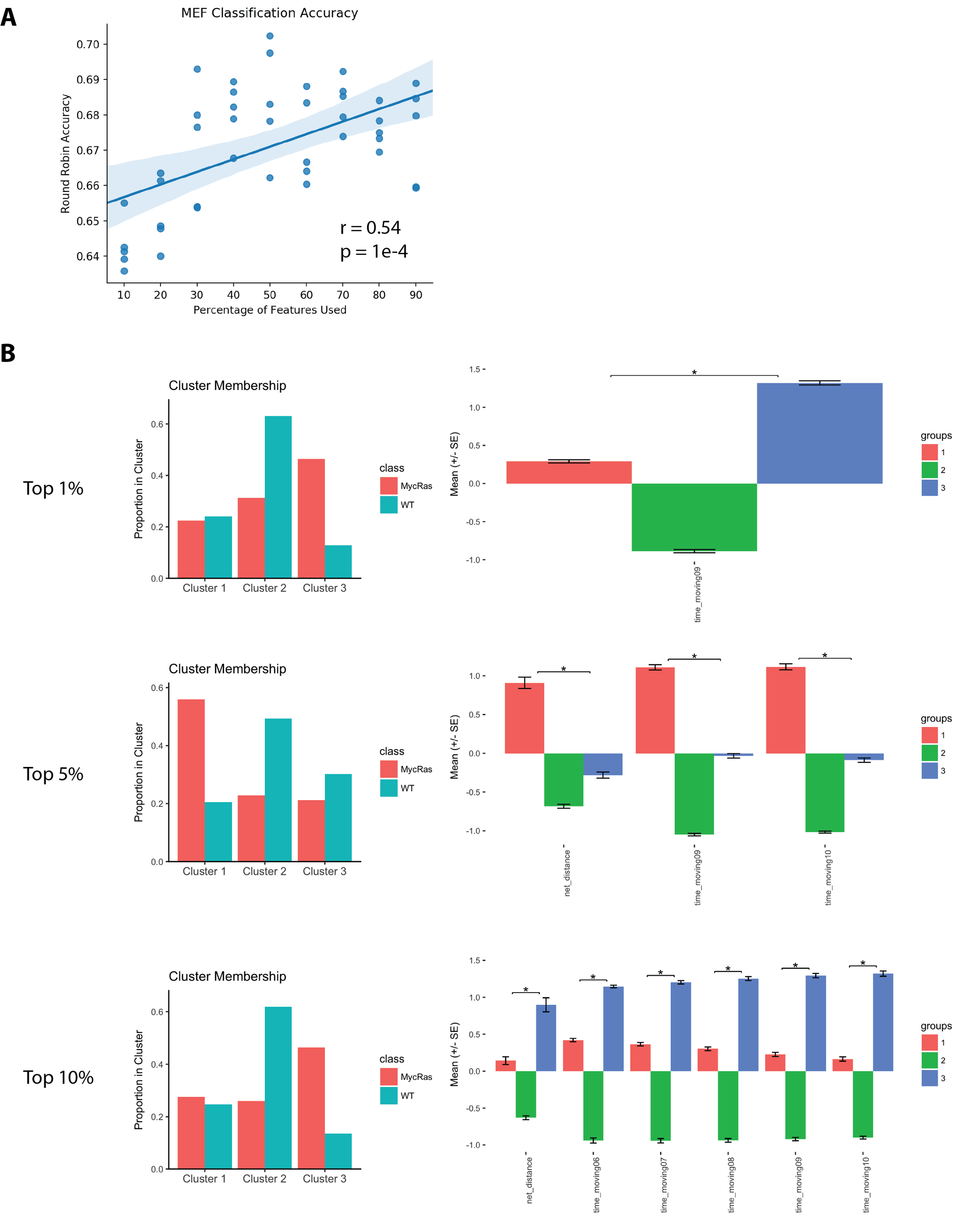

Supplement: S5 Fig — (A) Round Robin classification accuracy is significantly, positively correlated to the proportion of features utilized. Points represent mean Round Robin Classification Accuracy for a given parameter set of N% of features and a value for the SVM bias parameter C. Five bias parameter values were tested in a linear distribution in the range [0.4, 0.6] around the bias parameter C ≈ 0.5 we found for the optimal SVM by Grid Search. Reduced feature sets were selected using only the top N% of features based on ANOVA F-scores. (B) Cluster distributions and cluster feature values for clustering of MEFs with reduced feature sets. Feature sets were reduced to the top N% based on ANOVA F-scores. Behavioral clusters are still identifiable and MycRas/wild-type dependent state distribution is preserved with a single feature. (TIF) [file pcbi.1005927.s006.tif]

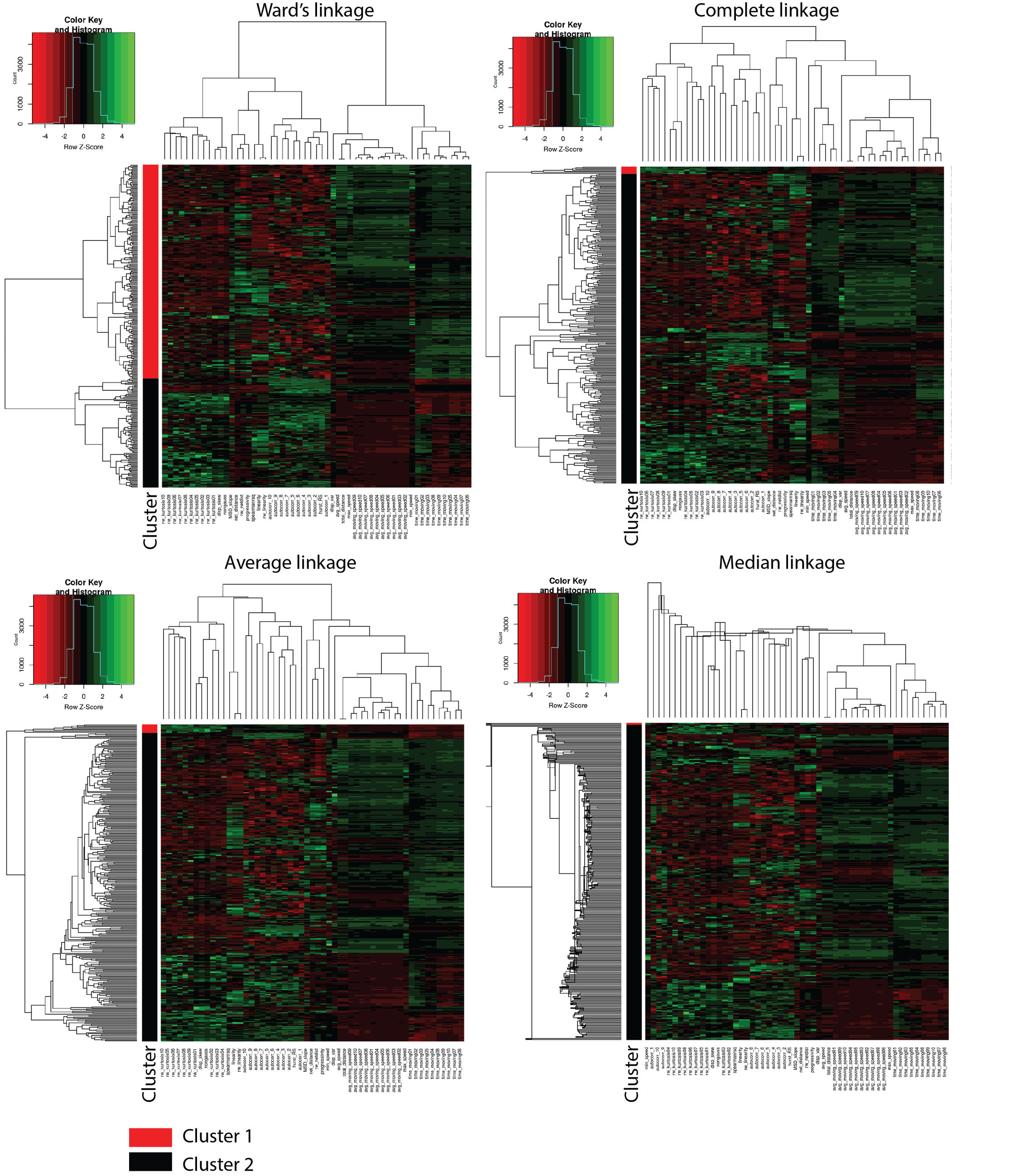

Supplement: S6 Fig — Clustergrams of myoblast feature space using several hierarchical clustering linkages. The assigned cluster label for each linkage map is displayed in a color coded column on the left hand side of each heatmap. Ward’s linkage was used for downstream analysis. (TIF) [file pcbi.1005927.s007.tif]

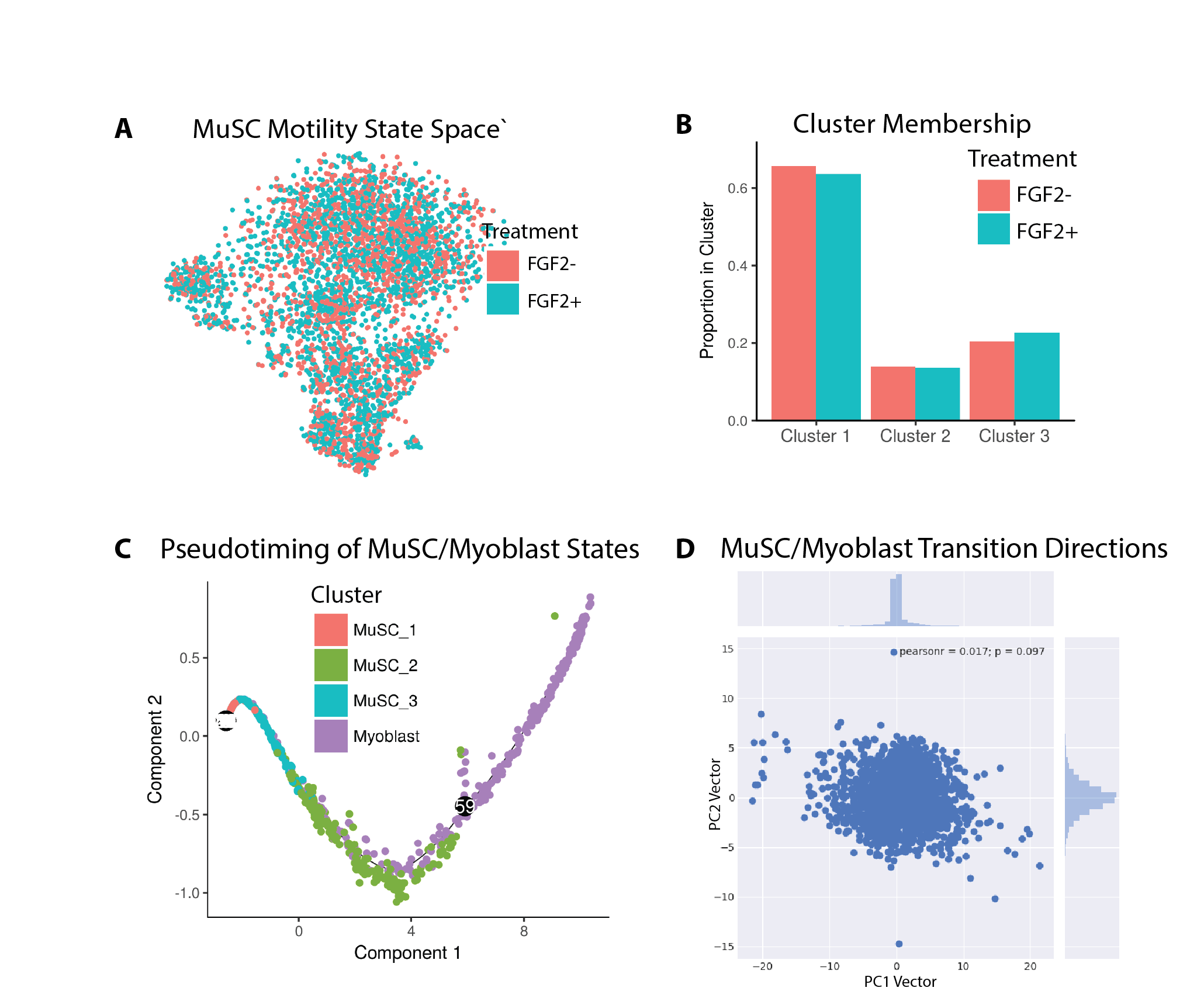

Supplement: S7 Fig — FGF2 does not influence MuSC motility phenotypes, and MuSC motility states reflect progressive states of activation. (A) t-SNE visualization of MuSC motility space with FGF2 treated and untreated color labels. (B) Occupancy of MuSC motility states in FGF2 treated and untreated conditions. (C) Pseudotime analysis displaying a reduced dimensional representation of MuSC and myoblast motility space (DDRT) with a minimum spanning tree plotted to mark the pseudotime axis. Pseudotime analysis attempts to find a temporal axis through an ergodic process observed at a single time point by fitting a minimum spanning tree (MST) to a reduced dimensional representation of multidimensional data. The longest axis of the MST is assumed to represent the temporal axis of the ergodic process. MuSC states are clearly ordered in a progressive sequence, moving toward the myoblast phenotype over pseudotime. (D) Scatterplot of MuSC/Myoblast transition vectors, demonstrating that transition vectors are primarily along the first principal component and slightly skewed in the positive direction. (TIF) [file pcbi.1005927.s008.tif]

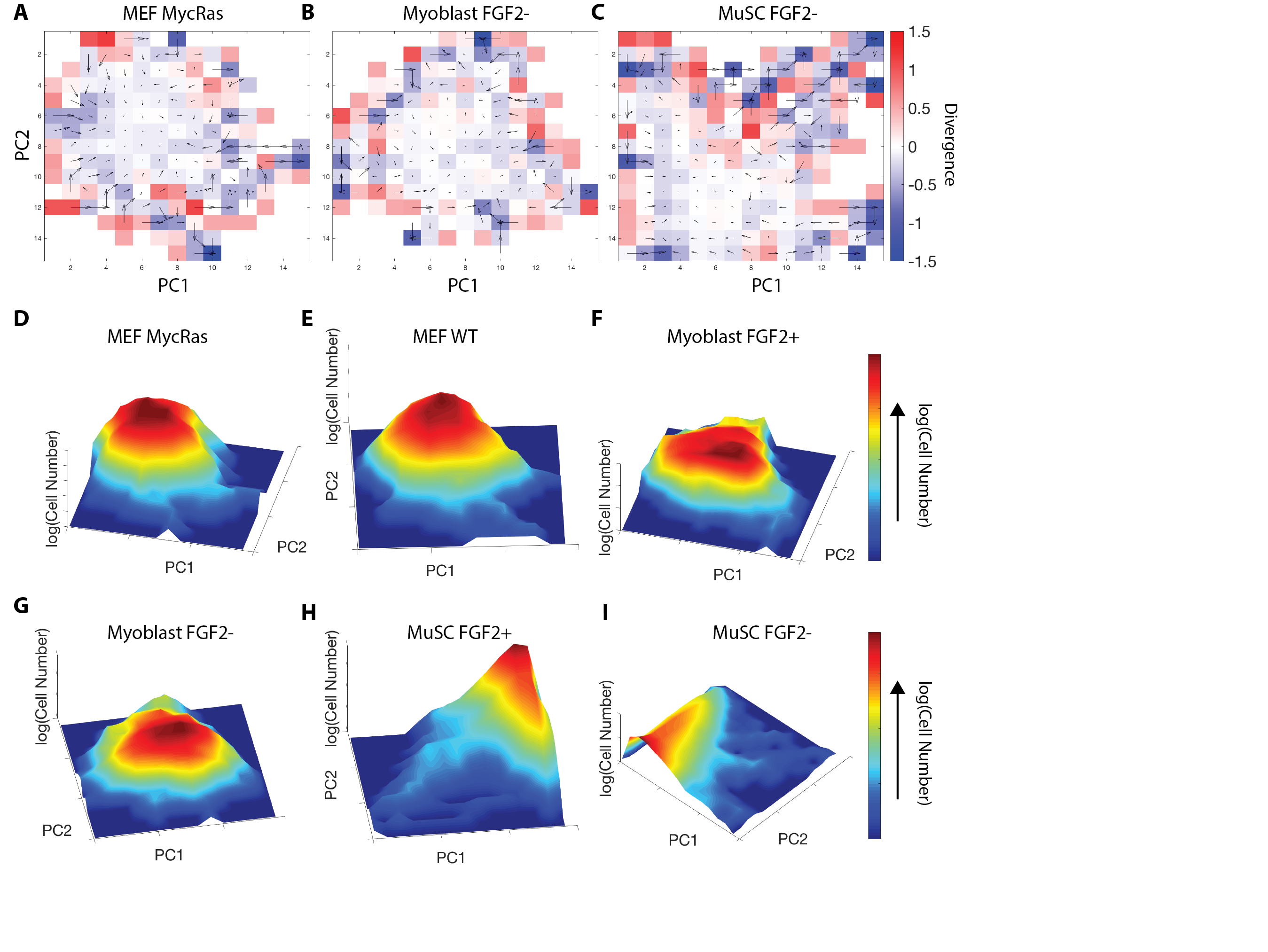

Supplement: S8 Fig — (A) Three-dimensional representation of the MycRas MEF state divergence surface as measured by cgPFA using tau = 20 and 15 course-grained bins. Course-grained probability flux analysis (cgPFA) of (B) myoblast (FGF2-), and (C) MuSC (FGF2+) motility states with subpaths of length τ = 20 time points (130 minutes) and 15 course-grained bins per dimension. Each unique combination of bins between PC1 and PC2 is considered as a unique state. Arrows represent transition rate vectors, calculated for each state bin as the vector mean of transitions into the neighboring states in the von Neumann neighborhood. Arrow direction represents the direction of these transition rate vectors, and arrow length represents transition rate vector magnitude. Underlying colors represent the vector divergence from that state as a metric of state stability. Positive divergence indicates cells are more likely to leave a state, while negative divergence indicates cells are more likely to enter a state. (D-I) State occupancy visualizations of the same course-grained PCA presented for cgPFA analysis. The number of cells that occupy a given state for at least one time unit is represented in the third dimension of the landscape and by the heatmap colors. (TIF) [file pcbi.1005927.s009.tif]

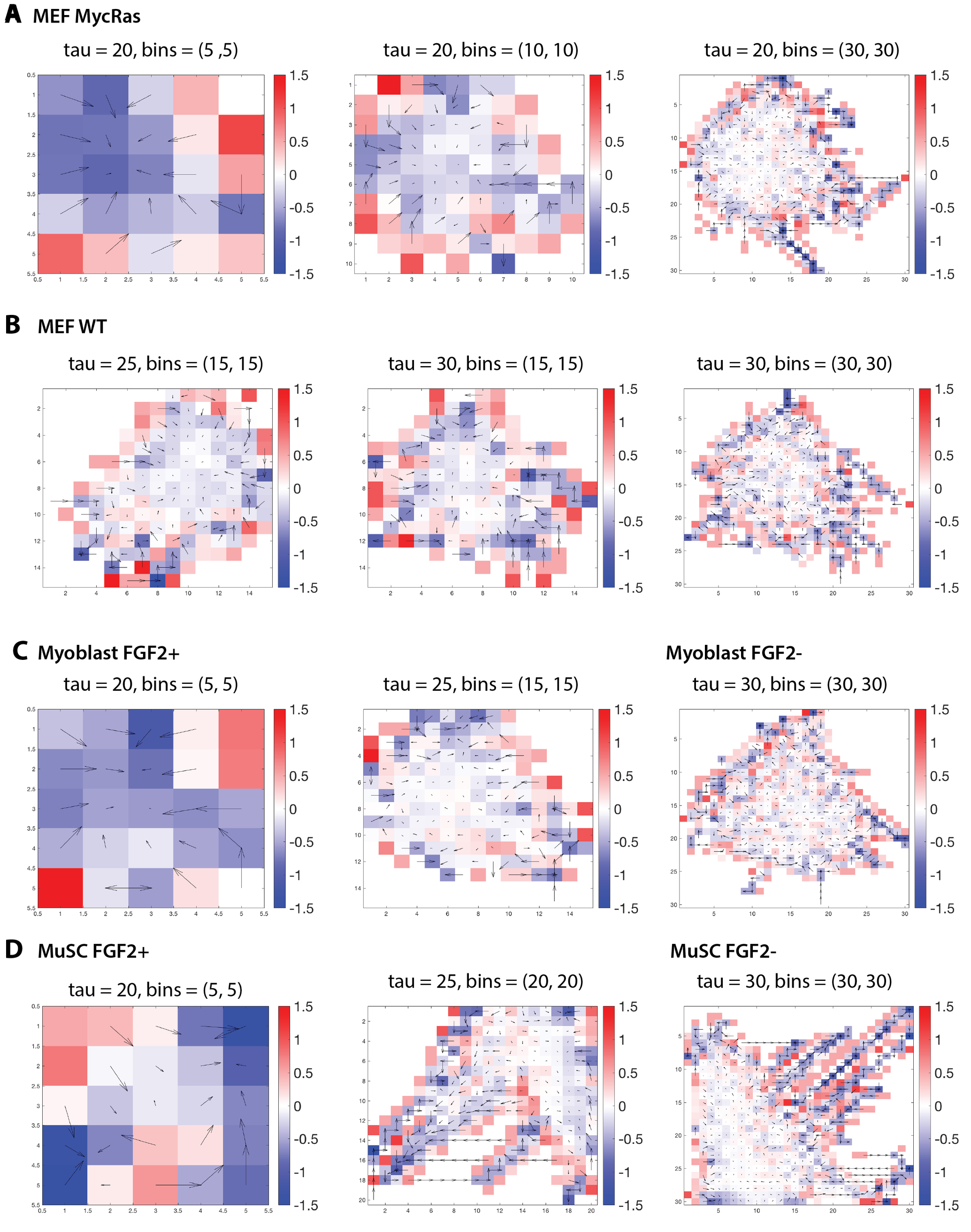

Supplement: S9 Fig — Course-grained PFA analysis as demonstrated in Fig 5 and S8 Fig was performed for all parameter combinations of the temporal window size tau ∈ {20, 25, 30} and binning resolution k ∈ {5, 10, 15, 20, 30} across all cellular systems. Representative visualizations across these parameter ranges are presented. Both (A) MycRas and (B) wild-type MEFs retain the qualitative metastable ‘basin’ appearance across time scales. As binning resolution decreases below k = 10, the structure of the state space is obscured. At higher resolutions of k, more bins little net divergence are present. (C) Myoblast cgPFA state spaces likewise retain a metastable ‘basin’ appearance across time scales. (D) MuSC state spaces retain a metastable ‘valley’ surrounded by unstable ridges across time scales. At higher binning resolutions of k, an unstable ridge within the metastable valley becomes more apparent. (TIF) [file pcbi.1005927.s010.tif]

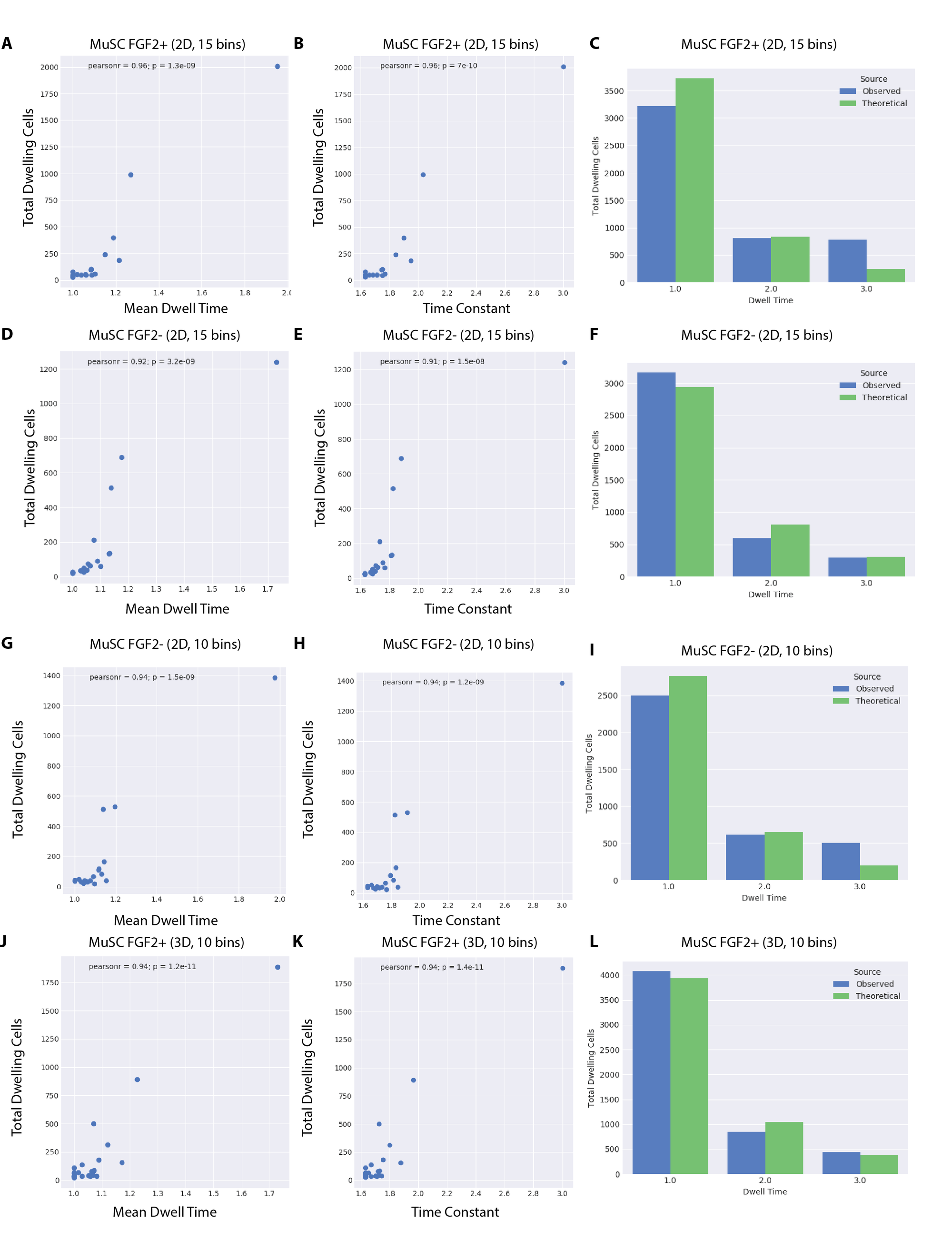

Supplement: S10 Fig — MuSC motility state dwell time analysis reveals rapid transitions, longer dwell times in higher occupancy states, and roughly exponentially distributed dwell times. Dwell times vs. total number of observed cells for each occupied state in course-grained PCA space for (A, B) FGF2- MuSCs, (D,E) FGF2+ MuSCs in two-dimensional PCA space, and (G, H) FGF2- MuSCs and (J, K) FGF2+ MuSCs in PCA spaces where detailed balance is broken. Dwell time distributions relative to the binned samples from a fitted exponential distribution for (C) FGF2- MuSCs and (F) FGF2+ MuSCs in two-dimensional PCA space, and (I) FGF2- MuSCs and (L) FGF2+ MuSCs in PCA space where detailed balance is broken. (TIF) [file pcbi.1005927.s011.tif]

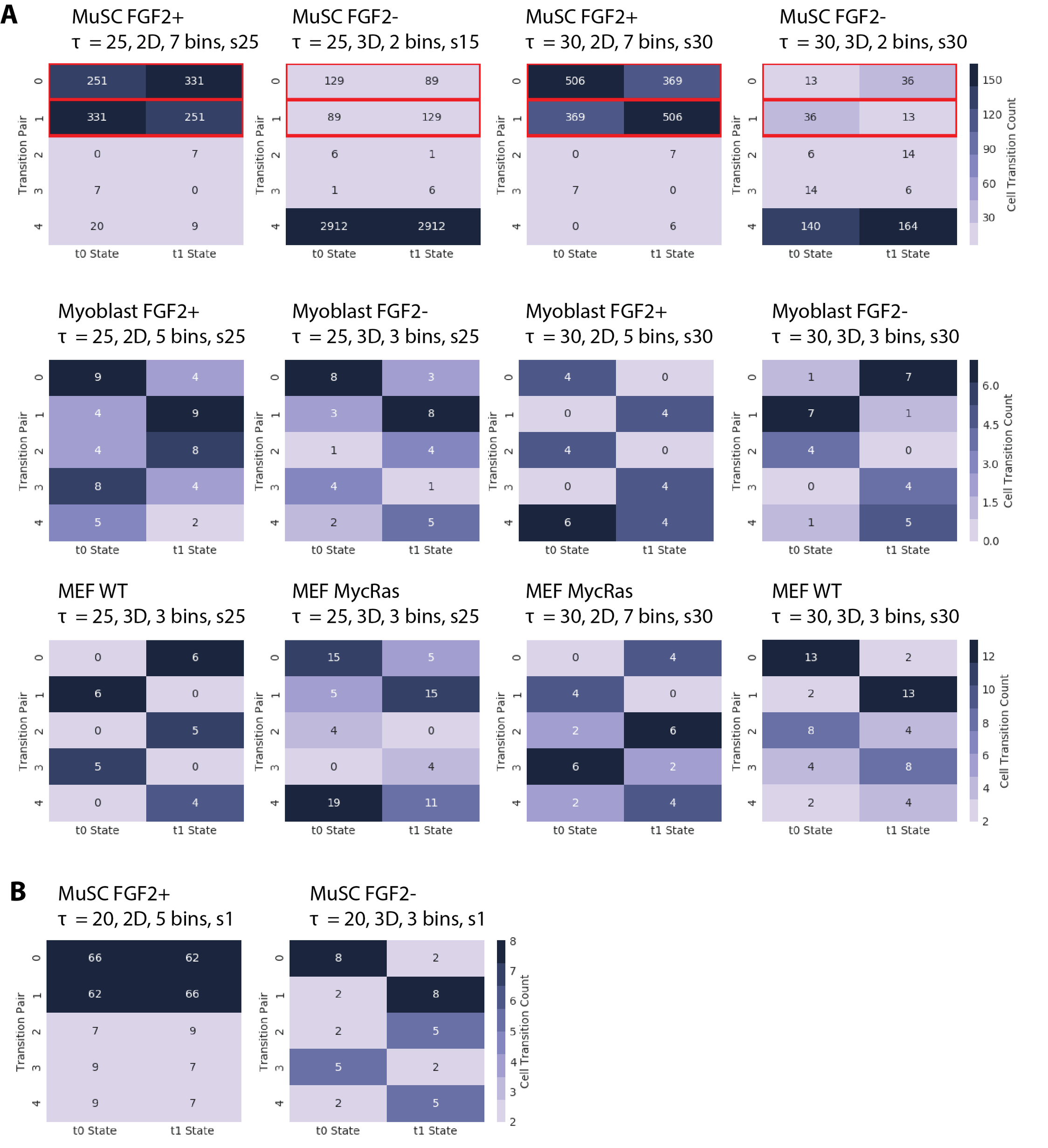

Supplement: S11 Fig — ND-cgPFA as presented in Fig 6 was repeated for values of the temporal window size parameter τ ∈ {20, 25, 30}. (A) The results of detailed balance breaking are robust across settings of this time scale parameter. At each time scale, the MuSC system breaks detailed balance, while the MEF and myoblast systems do not. Heatmaps display the five most unbalanced transitions for each defined cgPFA space. tau, course-grained bin, and stride parameters are listed above each heat map. (B) To demonstrate that detailed balance is present in the MuSC system on short time scales, we performed ND-cgPFA using the same number of temporal windows size tau = 20, but overlapped them with a single unit stride of s = 1. In this scheme, each window is only 1 time unit different than it’s neighbor, such that only 2 time units of difference are present between the initial and final time window. On this short time scale, MuSC systems do not break detailed balance. (TIF) [file pcbi.1005927.s012.tif]

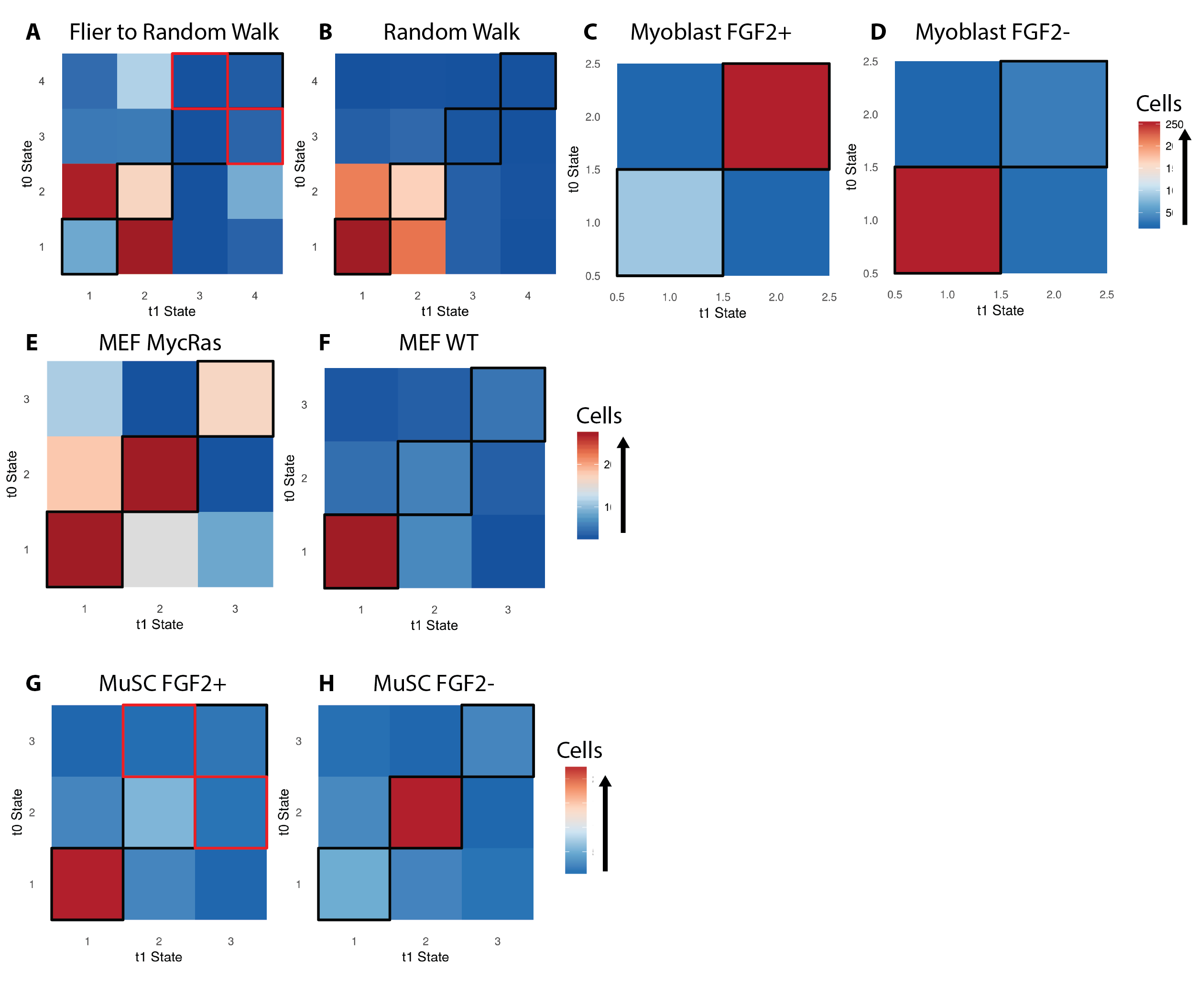

Supplement: S12 Fig — Hierarchical clustering based probability flux analysis of (A) a Levy flight simulation transitioning to a random walk, (B) an invariant random walk simulation, (C) myoblasts (FGF2+), (D) myoblasts (FGF2-), (E) MycRas MEFs, (F) WT MEFS, (G) MuSCs (FGF2+), and (H) MuSCs (FGF2-). The matrix displays transitions in state space as values in a matrix. Rows of the matrix correspond to an initial cell state (t0 state) and columns correspond to a destination state (t1 state). The value of each bin represents the number of times a state transition was observed. Symmetrical bins about the diagonal represent reciprocal pairwise transitions, with one ‘forward’ transition and one ‘reverse’ in each pair. The identity line represents “self” or non-transitions. MuSCs show a less balanced distribution than either MEFs or myoblasts by the binomial test for pairwise transition balance. Pairwise transitions breaking detailed balance are outlined in red. (TIF) [file pcbi.1005927.s013.tif]

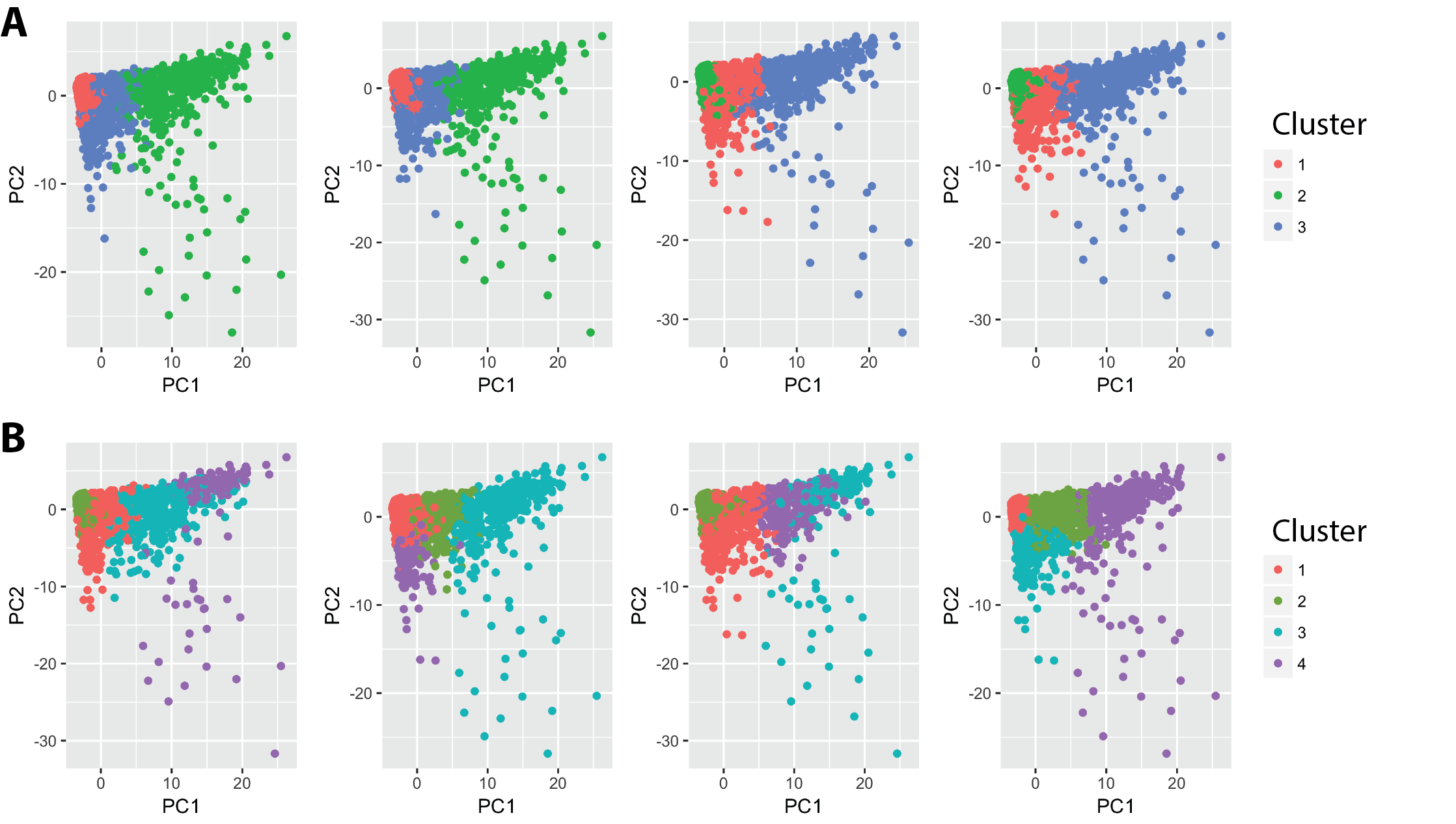

Supplement: S13 Fig — (A) Representative random samples of 80% of MuSCs with a 3 cluster partition (Ward’s linkage) applied. Note cluster separation along a common axis, robust to resampling. (B) Representative random samples of 80% of MuSCs with a 4 cluster partition (Ward’s linkage) applied. Note separation of clusters along multiple axes in some samples, and a single axis in others. The 4 cluster partition is not robust to resampling. (TIF) [file pcbi.1005927.s014.tif]
