# Supplementary material for: Inferring cell state by quantitative motility analysis reveals a dynamic state system and broken detailed balance
Source: PLoS Comput Biol. 2018 Jan 16;14(1):e1005927. doi: 10.1371/journal.pcbi.1005927 (PMC5786322; doi:10.1371/journal.pcbi.1005927)
Supplement: S1 Table — (PDF) [file pcbi.1005927.s021.pdf]

MuSC Cluster Indices

Cluster Number	KL	CH	Hartigan	CCC	Scott	Marriot	TrCovW	TraceW	Friedman	Rubin	Cindex	DB	Silhouette
2	10.177	2133.3919	312.3273	3.253	9562.851	8.33E+105	63922739	156054.9	6.4061	1.4945	0.1313	1.2232	0.5013
3	1.3637	1299.7855	229.4061	-3.869	14350.56	6.18E+105	51592520	145519.5	8.8867	1.6027	0.117	2.3348	0.1966
4	0.5982	988.8529	321.6707	-6.993	17769.284	4.98E+105	44302944	138170.3	11.3793	1.688	0.1138	1.9593	0.1955
5	0.7951	877.1795	400.7535	-0.7977	22125.234	2.83E+105	36537055	128578.4	14.6568	1.8139	0.1105	2.1395	0.198
6	4.7614	846.9323	118.7044	13.2222	28004.365	1.05E+105	28351105	117642.3	16.9323	1.9825	0.1392	1.7811	0.2026

Cluster Number	Frey	McClain	Dunn	Hubert	SDindex	Dindex	SDbw	Duda	Pseudot2	Beale	Ratkowsky	Ball	Ptbiserial
2	5.6984	0.1393	0.0637	0	0.5364	5.4054	0.8195	0.8924	448.9687	2.5492	0.0514	78027.43	0.7377
3	0.4698	0.5845	0.051	0	0.8803	5.1978	0.6703	0.8066	224.9506	5.0654	0.0876	48506.49	0.5336
4	-0.3418	0.624	0.051	0	0.7124	5.0617	0.5729	0.8351	116.3313	4.1691	0.0931	34542.57	0.5406
5	-0.5838	0.6246	0.051	0	0.834	4.9476	0.8162	0.6031	120.4471	13.8411	0.0984	25715.69	0.5618
6	10.027	0.623	0.0643	0	0.8511	4.866	0.8657	0.9361	189.8782	1.4421	0.1027	19607.06	0.5653

MEF Cluster Indices

Cluster Number	KL	CH	Hartigan	CCC	Scott	Marriot	TrCovW	TraceW	Friedman	Rubin	Cindex	DB	Silhouette
2	4.6302	171.6685	47.8417	-5.2284	777.5839	1.46E+79	1792136.8	23312.81	2.2141	1.3066	0.3047	1.8057	0.2031
3	1.1623	116.879	39.9319	-7.6527	1198.3601	1.56E+79	1384703.8	21477.91	3.2993	1.4182	0.3234	1.622	0.209
4	1.1531	96.623	34.6151	-8.2533	1590.2073	1.38E+79	1086941.6	20045.94	4.753	1.5195	0.3076	2.1997	0.1225
5	1.6694	85.463	22.9128	-7.781	2217.7564	7.05E+78	909495.6	18875.04	7.439	1.6137	0.2937	2.2809	0.0869
6	1.1785	75.6294	19.6934	-8.1122	2552.5809	5.59E+78	812386.1	18129.28	8.2825	1.6801	0.2886	2.1353	0.0846

Cluster Number	Frey	McClain	Dunn	Hubert	SDindex	Dindex	SDbw	Duda	Pseudot2	Beale	Ratkowsky	Ball	Ptbiserial
2	-0.2487	0.7808	0.1642	1.00E-04	0.5132	6.1704	0.5726	0.8449	48.11	3.8679	0.0558	11656.404	0.4
3	1.3961	0.8911	0.1816	1.00E-04	0.4418	5.977	0.5774	0.8753	42.1613	3.0016	0.0672	7159.305	0.4674
4	0.6426	1.5683	0.1589	1.00E-04	0.608	5.775	0.524	0.8605	37.6099	3.413	0.0786	5011.485	0.4207
5	0.4806	2.1752	0.1589	1.00E-04	0.645	5.6103	0.4729	0.8175	25.0001	4.678	0.0902	3775.008	0.4044
6	0.7461	2.4134	0.1589	1.00E-04	0.5852	5.4999	0.4363	0.8961	21.1115	2.4393	0.0946	3021.546	0.3999

Myoblast Cluster Indices

Cluster Number	KL	CH	Hartigan	CCC	Scott	Marriot	TrCovW	TraceW	Friedman	Rubin	Cindex	DB	Silhouette
2	5.3964	122.0694	29.2421	-1.5802	515.5641	2.53E+69	490843.7	11964.159	3.3863	1.3989	0.2603	1.5237	0.3147
3	1.098	81.2221	25.9006	-3.3977	924.6677	1.51E+69	393087.4	10920.564	5.2256	1.5326	0.2493	1.5746	0.2998
4	1.0359	67.1589	24.9802	-3.6226	1177.1696	1.18E+69	305487.9	10065.779	7.138	1.6628	0.2373	2.1118	0.1162
5	1.4099	60.5533	18.8135	-2.4975	1590.4825	4.82E+68	246283.6	9301.462	11.4558	1.7994	0.2264	2.0435	0.1081

Cluster Number	Frey	McClain	Dunn	Hubert	SDindex	Dindex	SDbw	Duda	Pseudot2	Beale	Ratkowsky	Ball	Ptbiserial
2	-0.4129	0.4435	0.1387	2.00E-04	0.5256	5.8627	0.6352	0.8046	20.8827	5.0753	0.0542	5982.08	0.5896
3	5.9771	0.466	0.1387	2.00E-04	0.6259	5.6625	0.8719	0.8709	32.3063	3.1191	0.0829	3640.188	0.6409
4	0.1043	1.4458	0.1254	2.00E-04	0.8245	5.4227	0.709	0.7551	23.3551	6.7648	0.0918	2516.445	0.4335
5	-0.2244	1.5856	0.1254	3.00E-04	0.7472	5.2301	0.603	0.5381	10.3017	16.7555	0.103	1860.292	0.4505
