# Supplementary material for: Inferring cell state by quantitative motility analysis reveals a dynamic state system and broken detailed balance
Source: PLoS Comput Biol. 2018 Jan 16;14(1):e1005927. doi: 10.1371/journal.pcbi.1005927 (PMC5786322; doi:10.1371/journal.pcbi.1005927)
Supplement: S2 Table — (PDF) [file pcbi.1005927.s022.pdf]

Variation Group	brw_bias	brw_mu	fbm_H	pf_alpha
1	0.99	20	0.99	5
2	0.99	20	0.99	5
3	0.99	5	0.1	5
4	0.99	5	0.1	0.5
5	0.7	5	0.99	5
6	0.1	5	0.99	5
7	0.7	20	0.1	5
8	0.7	5	0.99	5
9	0.1	20	0.5	5
10	0.1	20	0.5	5

urw_mu	Unsupervised Clustering Acc.
50	1
10	1
10	1
50	0.997
20	0.99975
20	0.902
10	0.9965
50	1
20	0.83925
10	0.997
